# Molecular states during acute COVID-19 reveal distinct etiologies of long-term sequelae

**DOI:** 10.1038/s41591-022-02107-4

**Published:** 2022-12-08

**Authors:** Ryan C. Thompson, Nicole W. Simons, Lillian Wilkins, Esther Cheng, Diane Marie Del Valle, Gabriel E. Hoffman, Carlo Cervia, Brian Fennessy, Konstantinos Mouskas, Nancy J. Francoeur, Jessica S. Johnson, Lauren Lepow, Jessica Le Berichel, Christie Chang, Aviva G. Beckmann, Ying-chih Wang, Kai Nie, Nicholas Zaki, Kevin Tuballes, Vanessa Barcessat, Mario A. Cedillo, Dan Yuan, Laura Huckins, Panos Roussos, Thomas U. Marron, The Mount Sinai COVID-19 Biobank Team, Charuta Agashe, Priyal Agrawal, Alara Akyatan, Kasey Alesso-Carra, Eziwoma Alibo, Kelvin Alvarez, Angelo Amabile, Carmen Argmann, Kimberly Argueta, Steven Ascolillo, Rasheed Bailey, Craig Batchelor, Noam D. Beckmann, Priya Begani, Dusan Bogunovic, Swaroop Bose, Cansu Cimen Bozkus, Paloma Bravo, Stacey-Ann Brown, Mark Buckup, Larissa Burka, Sharlene Calorossi, Lena Cambron, Guillermo Carbonell, Gina Carrara, Mario A. Cedillo, Christie Chang, Serena Chang, Steven T. Chen, Jonathan Chien, Mashkura Chowdhury, Jonathan Chung, Phillip H. Comella, Dana Cosgrove, Francesca Cossarini, Liam Cotter, Arpit Dave, Travis Dawson, Bheesham Dayal, Maxime Dhainaut, Rebecca Dornfeld, Katie Dul, Melody Eaton, Nissan Eber, Cordelia Elaiho, Ethan Ellis, Frank Fabris, Jeremiah Faith, Dominique Falci, Susie Feng, Marie Fernandes, Nataly Fishman, Nancy J. Francoeur, Sandeep Gangadharan, Daniel Geanon, Bruce D. Gelb, Benjamin S. Glicksberg, Sacha Gnjatic, Edgar Gonzalez-Kozlova, Joanna Grabowska, Gavin Gyimesi, Maha Hamdani, Diana Handler, Jocelyn Harris, Matthew Hartnett, Sandra Hatem, Manon Herbinet, Elva Herrera, Arielle Hochman, Gabriel E. Hoffman, Jaime Hook, Laila Horta, Etienne Humblin, Suraj Jaladanki, Hajra Jamal, Daniel Jordan, Gurpawan Kang, Neha Karekar, Subha Karim, Geoffrey Kelly, Jong Kim, Seunghee Kim-Schulze, Arvind Kumar, Jose Lacunza, Alona Lansky, Dannielle Lebovitch, Brian Lee, Grace Lee, Gyu Ho Lee, Jacky Lee, John Leech, Michael B. Leventhal, Lora E. Liharska, Katherine Lindblad, Alexandra Livanos, Rosalie Machado, Kent Madrid, Zafar Mahmood, Kelcey Mar, Thomas U. Marron, Glenn Martin, Robert Marvin, Shrisha Maskey, Paul Matthews, Katherine Meckel, Saurabh Mehandru, Miriam Merad, Cynthia Mercedes, Elyze Merzier, Dara Meyer, Gurkan Mollaoglu, Sarah Morris, Konstantinos Mouskas, Emily Moya, Girish Nadkarni, Kai Nie, Marjorie Nisenholtz, George Ofori-Amanfo, Kenan Onel, Merouane Ounadjela, Manishkumar Patel, Vishwendra Patel, Cassandra Pruitt, Adeeb Rahman, Shivani Rathi, Jamie Redes, Ivan Reyes-Torres, Alcina Rodrigues, Alfonso Rodriguez, Vladimir Roudko, Panos Roussos, Evelyn Ruiz, Pearl Scalzo, Eric E. Schadt, Ieisha Scott, Robert Sebra, Sandra Serrano, Hardik Shah, Mark Shervey, Pedro Silva, Laura Sloofman, Melissa Smith, Alessandra Soares Schanoski, Juan Soto, Shwetha Hara Sridhar, Hiyab Stefanos, Meghan Straw, Robert Sweeney, Alexandra Tabachnikova, Collin Teague, Manying Tin, Kevin Tuballes, Scott R. Tyler, Bhaskar Upadhyaya, Akhil Vaid, Verena Van Der Heide, Natalie Vaninov, Konstantinos Vlachos, Daniel Wacker, Laura Walker, Hadley Walsh, Bo Wang, Wenhui Wang, Ying-chih Wang, C. Matthias Wilk, Jessica Wilson, Karen M. Wilson, Hui Xie, Li Xue, Naa-akomaah Yeboah, Nancy Yi, Mahlet Yishak, Sabina Young, Alex Yu, Nicholas Zaki, Nina Zaks, Renyuan Zha, Benjamin S. Glicksberg, Girish Nadkarni, James R. Heath, Edgar Gonzalez-Kozlova, Onur Boyman, Seunghee Kim-Schulze, Robert Sebra, Miriam Merad, Sacha Gnjatic, Eric E. Schadt, Alexander W. Charney, Noam D. Beckmann

**Affiliations:** 1 Mount Sinai Clinical Intelligence Center, Icahn School of Medicine at Mount Sinai, New York, NY USA; 2 Charles Bronfman Institute for Personalized Medicine, Icahn School of Medicine at Mount Sinai, New York, NY USA; 3 Icahn School of Medicine at Mount Sinai, New York, NY USA; 4 Precision Immunology Institute, Icahn School of Medicine at Mount Sinai, New York, NY USA; 5 Department of Genetics and Genomic Sciences, Pamela Sklar Division of Psychiatric Genomics, Icahn School of Medicine at Mount Sinai, New York, NY USA; 6 Department of Immunology, University Hospital Zurich, University of Zurich, Zurich, Switzerland; 7 Susan and Leonard Feinstein Inflammatory Bowel Disease Clinical Center, Icahn School of Medicine at Mount Sinai, New York, NY USA; 8 Department of Genetics and Genomic Sciences, Icahn School of Medicine at Mount Sinai, New York, NY USA; 9 Center for Advanced Genomics Technology, Icahn School of Medicine at Mount Sinai, New York, NY USA; 10 Human Immune Monitoring Center, Icahn School of Medicine at Mount Sinai, New York, NY USA; 11 Sema4, a Mount Sinai venture, Stamford, CT USA; 12 Department of Diagnostic, Molecular and Interventional Radiology, Icahn School of Medicine at Mount Sinai, New York, NY USA; 13 Institute for Systems Biology, Seattle, WA USA; 14 Department of Bioengineering, University of Washington, Seattle, WA USA; 15 Department of Psychiatry, Icahn School of Medicine at Mount Sinai, New York, NY USA; 16 Center for Disease Neurogenomics, Icahn School of Medicine at Mount Sinai, New York, NY USA; 17 Friedman Brain Institute, Icahn School of Medicine at Mount Sinai, New York, NY USA; 18 Icahn Institute for Data Science and Genomic Technology, Icahn School of Medicine at Mount Sinai, New York, NY USA; 19 Mental Illness Research Education and Clinical Center (VISN 2 South), James J. Peters VA Medical Center, Bronx, NY USA; 20 Center for Dementia Research, Nathan Kline Institute for Psychiatric Research, Orangeburg, NY USA; 21 Tisch Cancer Institute, Icahn School of Medicine at Mount Sinai, New York, NY USA; 22 Department of Medicine, Icahn School of Medicine at Mount Sinai, New York, NY USA; 23 Department of Medicine, Division of Hematology and Oncology, Icahn School of Medicine at Mount Sinai, New York, NY USA; 24 Hasso Plattner Institute for Digital Health at Mount Sinai, Icahn School of Medicine at Mount Sinai, New York, NY USA; 25 Department of Medicine, Division of Data Driven and Digital Medicine (D3M), Icahn School of Medicine at Mount Sinai, New York, NY USA; 26 Faculty of Medicine, University of Zurich, Zurich, Switzerland; 27 Department of Oncological Sciences, Icahn School of Medicine at Mount Sinai, New York, NY USA; 28 Black Family Stem Cell Institute, Icahn School of Medicine at Mount Sinai, New York, NY USA

**Keywords:** Gene ontology, Infectious diseases, Transcriptomics, Viral infection, Gene regulation in immune cells

## Abstract

Post-acute sequelae of severe acute respiratory syndrome coronavirus 2 (SARS-CoV-2) infection are debilitating, clinically heterogeneous and of unknown molecular etiology. A transcriptome-wide investigation was performed in 165 acutely infected hospitalized individuals who were followed clinically into the post-acute period. Distinct gene expression signatures of post-acute sequelae were already present in whole blood during acute infection, with innate and adaptive immune cells implicated in different symptoms. Two clusters of sequelae exhibited divergent plasma-cell-associated gene expression patterns. In one cluster, sequelae associated with higher expression of immunoglobulin-related genes in an anti-spike antibody titer-dependent manner. In the other, sequelae associated independently of these titers with lower expression of immunoglobulin-related genes, indicating lower non-specific antibody production in individuals with these sequelae. This relationship between lower total immunoglobulins and sequelae was validated in an external cohort. Altogether, multiple etiologies of post-acute sequelae were already detectable during SARS-CoV-2 infection, directly linking these sequelae with the acute host response to the virus and providing early insights into their development.

## Abstract (web summary)

Transcriptomic analyses of acute phase whole blood from a large cohort of patients with COVID-19 identify molecular determinants of post-infection long-term sequelae.

## Main

Since the outbreak of severe acute respiratory syndrome coronavirus 2 (SARS-CoV-2), over 480 million individuals have developed Coronavirus Disease 2019 (COVID-19). The post-acute sequelae of SARS-CoV-2 infection (PASC) comprise a broad array of symptoms that emerge after recovery in over half of COVID-19 survivors^1–3^. PASC symptoms include fatigue, dyspnea and smell/taste problems, often lasting over long periods of time^4–7^. The acute phase of COVID-19 (described hereafter as ‘acute’) has been reported to be associated with certain PASC outcomes through elements of the immune response to SARS-CoV-2 infection^7–15^. However, examined sample sizes have often been small and the scope of molecular profiling generally limited.

Two recent studies have interrogated the immunology of PASC, especially as it relates to acute COVID-19, with larger cohorts comprising both hospitalized and non-hospitalized patients, and using broader molecular profiling^16,17^. No significant association was observed between PASC and acute titers of antibodies against the SARS-CoV-2 spike surface protein (anti-spike antibodies)^17,18^. In contrast, decreased total acute antibody (immunoglobulin) titers were found to predict the development of any PASC symptoms^17^. Different subsets of PASC symptoms were also associated with acute measures derived from multi-omics data of SARS-CoV-2 RNA in blood, presence of Epstein–Barr virus, distinct CD8^+^ and CD4^+^ T cell phenotypes and autoantibodies^16^. PASC was also associated with post-acute detection of autoantibodies, as were several subsets of PASC symptoms^16,19^. Multiple hypotheses have been proposed to connect acute COVID-19 and PASC, including chronic inflammation driven by persisting viral reservoirs, autoimmunity, dysbiosis of microbiome or virome and long-lasting tissue damage^20^. Although these studies identify an array of acute risk factors for PASC, there remains a need for more comprehensive characterization of the heterogeneous molecular processes of the acute host response to SARS-CoV-2 infection that associate with subsequent development of PASC.

In this study, whole blood gene expression and antibody titers were profiled in a large cohort of hospitalized patients with COVID-19 who were followed clinically into the post-acute period^21^. Distinct acute phase cell-type-specific (CTS) gene expression signatures were identified linking several immune cell types to post-acute sequelae 1 year after discharge. At least two independent etiologies of PASC were identified, distinguished by their dependence on anti-spike antibody titers. Together, our results reveal that the molecular processes leading to PASC are already detectable during acute COVID-19, establish multiple distinct etiologies leading to different long-term outcomes and directly link the emergence of these symptoms to the host response to SARS-CoV-2 infection.

## Results

### Limited association of symptoms with anti-spike antibodies

**Fig. 1:**
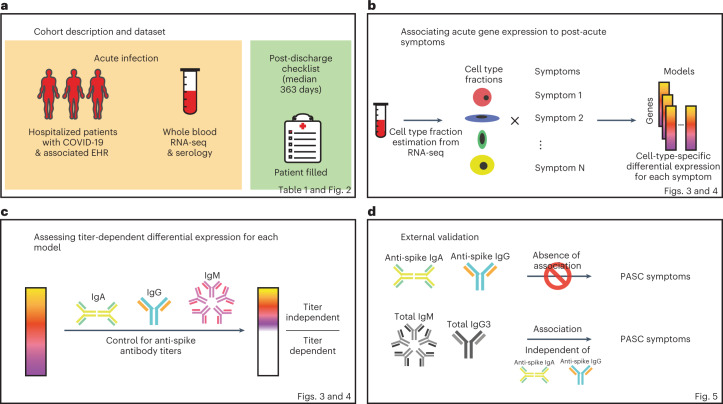
Study workflow. Schematics of the study design, analysis workflow and validation. **a**, Summary of the cohort studied and data collected. **b**, Strategy for CTS differential expression testing for PASC symptoms. **c**, Strategy for distinguishing DEGs by whether or not their differential expression is dependent on anti-spike antibody titers. **d**, Validation strategies employed using independent external datasets.

**Fig. 2:**
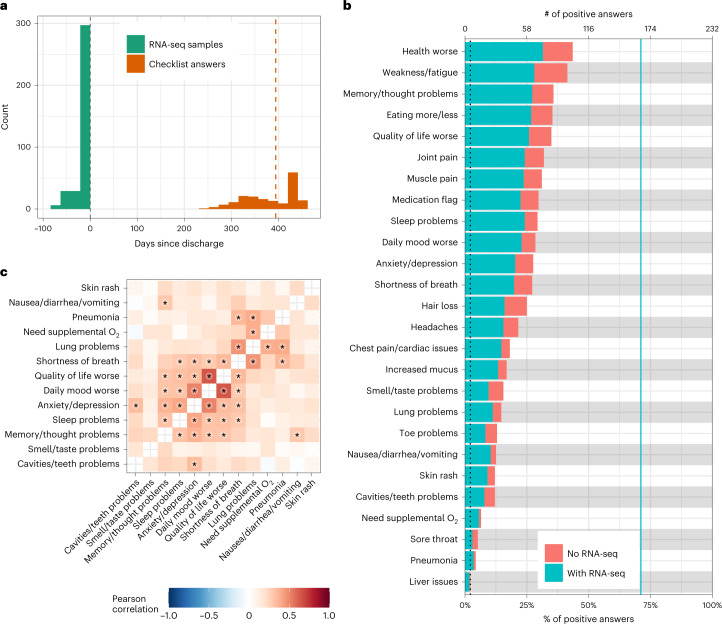
Description of PASC symptoms. **a**, Histogram of the timing of blood sampling and PASC checklist completion. The *x* and *y* axes are the number of days since discharge and a count of observations, respectively. The green bars are counts of RNA-seq samples, and the orange bars represent the number of days between COVID-19 hospitalization discharge (black dashed line) and PASC checklist completion (dashed orange line is the median). **b**, Prevalence of PASC symptoms in our cohort. The *y* axis is symptoms, and the upper and lower *x* axes are the number of positive answers and percentage of individuals from the entire cohort with a positive answer, respectively. The blue line represents the subset of individuals with RNA-seq who completed the checklist. The dashed black line is the cutoff used for inclusion in follow-up analyses. **c**, PASC checklist item correlations. The axes are representative of the symptoms of interest (Methods), and the color is the Pearson correlation of their coincidence. Correlations with FWER (Holm’s method) adjusted *P* < 0.05 (two-sided Fisher’s exact test) are indicated with a star. Rows and columns are ordered to minimize distance between adjacent symptoms. Source data

**Extended Data Fig. 1:**
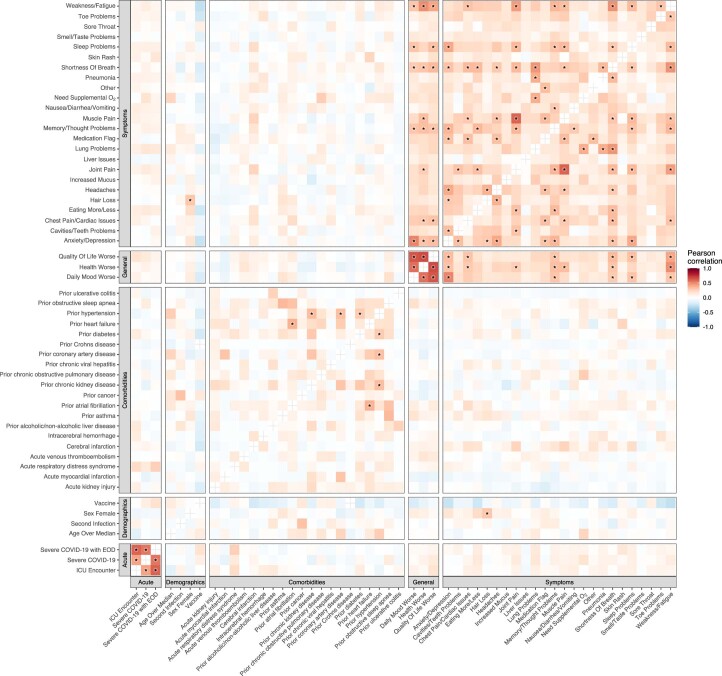
Correlation of occurrences of PASC checklist items, comorbidities, demographics, and acute disease metrics. The axes are representative of the symptoms, comorbidities, demographics, and acute disease metrics, and the color represents the Pearson correlation of their coincidence. Comorbidities present before COVID-19 hospitalization are defined with the prefix ‘prior’ in the axis label. Correlations with family wise error rate (FWER, Holm’s method) adjusted *P* values < 0.05 (2-sided Fisher’s exact test) are indicated with a star. Rows and columns are ordered by hierarchical clustering and optimal leaf ordering. Source data

In this study, 567 individuals (495 hospitalized with COVID-19 and 72 healthy and hospitalized controls) were enrolled in the Mount Sinai COVID-19 Biobank Study between April and June 2020 (Fig. 1). Blood was collected from hospitalized individuals serially throughout their stay and from healthy controls at a single timepoint in the outpatient setting, and RNA-sequencing (RNA-seq) was generated from these (*n* = 1,392). Six months or more after discharge from COVID-19 hospitalization (median = 363 days), 232 individuals (165 with RNA-seq) completed a self-reported checklist assessing for the emergence of PASC (Table 1, Supplementary Table 1a,b and Fig. 2a,b; checklist items referred to as symptoms). No symptoms were significantly associated with having received any vaccine dose among the 50 individuals whose date of first SARS-CoV-2 vaccine was known (Extended Data Fig. 1). The effect of SARS-CoV-2 reinfections on symptom prevalence could not be assessed owing to the low number of documented reinfections before checklist completion (*n* = 14, not enough statistical power).

**Table 1:** Cohort description

	Overall		Any PASC symptom		No PASC symptoms	
	Full cohort (*n* = 232)	With RNA (*n* = 165)	Full cohort (*n* = 195)	With RNA (*n* = 140)	Full cohort (*n* = 37)	With RNA (*n* = 25)
Demographics						
Female	97 (42%)	75 (45%)	87 (45%)	66 (47%)	10 (27%)	9 (36%)
Age	58 ± 16 (19–90)	60 ± 16 (22–90)	58 ± 16 (22–90)	60 ± 17 (22–90)	56 ± 16 (19–88)	59 ± 14 (26–88)
Race: American Indian/Alaska Native	3 (1%)	2 (1%)	2 (1%)	1 (1%)	1 (3%)	1 (4%)
Race: Asian	25 (11%)	21 (13%)	22 (11%)	18 (13%)	3 (8%)	3 (12%)
Race: Black or African American	56 (24%)	40 (24%)	46 (24%)	34 (24%)	10 (27%)	6 (24%)
Race: More Than One Race	16 (7%)	9 (5%)	15 (8%)	8 (6%)	1 (3%)	1 (4%)
Race: Native Hawaiian or Other Pacific Islander	1 (0%)	1 (1%)	1 (1%)	1 (1%)	0 (0%)	0 (0%)
Race: Unknown/Prefer not to say	41 (18%)	36 (22%)	31 (16%)	28 (20%)	10 (27%)	8 (32%)
Race: White	89 (38%)	56 (34%)	77 (39%)	50 (36%)	12 (32%)	6 (24%)
Race: (Not reported)	1 (0%)	0 (0%)	1 (1%)	0 (0%)	0 (0%)	0 (0%)
Ethnicity: Hispanic or Latino	67 (29%)	54 (33%)	56 (29%)	45 (32%)	11 (30%)	9 (36%)
Ethnicity: Not Hispanic or Latino	161 (69%)	108 (65%)	136 (70%)	93 (66%)	25 (68%)	15 (60%)
Ethnicity: Unknown/Prefer not to say	3 (1%)	3 (2%)	2 (1%)	2 (1%)	1 (3%)	1 (4%)
Ethnicity: (Not reported)	1 (0%)	0 (0%)	1 (1%)	0 (0%)	0 (0%)	0 (0%)
Acute COVID-19 clinical characteristics						
Severe COVID-19	53 (23%)	39 (24%)	44 (23%)	33 (24%)	9 (24%)	6 (24%)
Severe COVID-19 with EOD	37 (16%)	31 (19%)	35 (18%)	30 (21%)	2 (5%)	1 (4%)
ICU	54 (23%)	34 (21%)	45 (23%)	31 (22%)	9 (24%)	3 (12%)
Comorbidities						
Any comorbidity	132 (57%)	115 (70%)	114 (58%)	101 (72%)	18 (49%)	14 (56%)
Acute respiratory distress syndrome	3 (1%)	3 (2%)	3 (2%)	3 (2%)	0 (0%)	0 (0%)
Acute kidney injury	17 (7%)	15 (9%)	12 (6%)	11 (8%)	5 (14%)	4 (16%)
Acute venous thromboembolism	2 (1%)	2 (1%)	2 (1%)	2 (1%)	0 (0%)	0 (0%)
Acute cerebral infarction	5 (2%)	4 (2%)	5 (3%)	4 (3%)	0 (0%)	0 (0%)
Acute myocardial infarction	1 (0%)	1 (1%)	1 (1%)	1 (1%)	0 (0%)	0 (0%)
Prior asthma	15 (6%)	13 (8%)	14 (7%)	12 (9%)	1 (3%)	1 (4%)
Prior chronic obstructive pulmonary disease	11 (5%)	11 (7%)	9 (5%)	9 (6%)	2 (5%)	2 (8%)
Prior hypertension	83 (36%)	75 (45%)	70 (36%)	63 (45%)	13 (35%)	12 (48%)
Prior obstructive sleep apnea	12 (5%)	10 (6%)	12 (6%)	10 (7%)	0 (0%)	0 (0%)
Prior diabetes	54 (23%)	47 (28%)	47 (24%)	42 (30%)	7 (19%)	5 (20%)
Prior chronic kidney disease	28 (12%)	25 (15%)	24 (12%)	22 (16%)	4 (11%)	3 (12%)
Prior cancer	22 (9%)	20 (12%)	19 (10%)	18 (13%)	3 (8%)	2 (8%)
Prior coronary artery disease	26 (11%)	23 (14%)	21 (11%)	18 (13%)	5 (14%)	5 (20%)
Prior atrial fibrillation	18 (8%)	16 (10%)	18 (9%)	16 (11%)	0 (0%)	0 (0%)
Prior heart failure	17 (7%)	15 (9%)	14 (7%)	13 (9%)	3 (8%)	2 (8%)
Prior chronic viral hepatitis	3 (1%)	2 (1%)	3 (2%)	2 (1%)	0 (0%)	0 (0%)
Prior alcoholic/non-alcoholic liver disease	6 (3%)	3 (2%)	5 (3%)	3 (2%)	1 (3%)	0 (0%)
Prior Crohnʼs disease	2 (1%)	1 (1%)	2 (1%)	1 (1%)	0 (0%)	0 (0%)
Prior ulcerative colitis	1 (0%)	0 (0%)	0 (0%)	0 (0%)	1 (3%)	0 (0%)

Numerical variables are shown as mean ± standard deviation (minimum–maximum). Categorical variables are shown as total number (percent). Data are shown for the full cohort that provided answers to the PASC checklist items, the full cohort that provided answers to the PASC checklist items with any PASC sequelae and the full cohort that provided answers to the PASC checklist items without any PASC sequelae. For each cohort, population characteristics are provided for all individuals in the cohort as well as for the subset with RNA-seq. Further characterizations of the cohorts can be found in Supplementary Table 1a,b.

**Extended Data Fig. 2:**
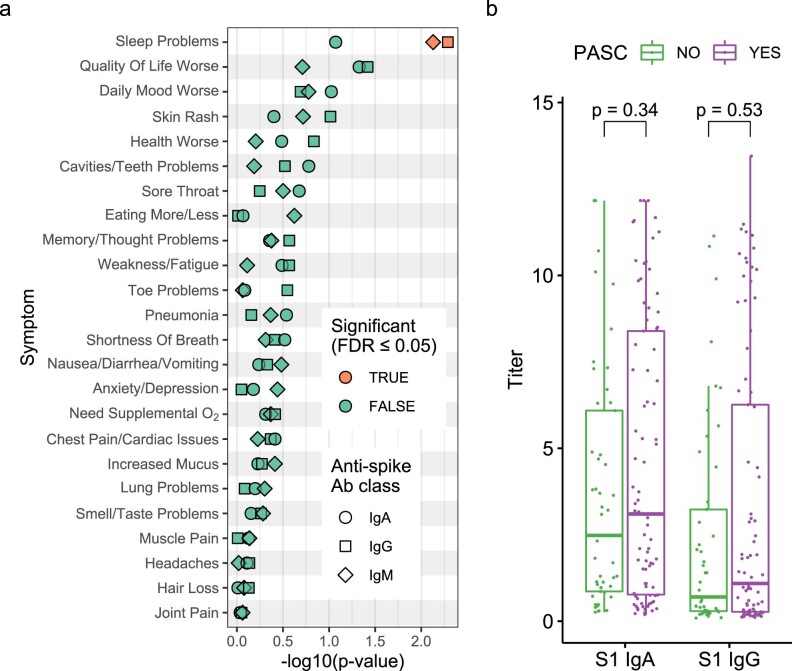
Relationship between acute anti-spike antibody titers and PASC with independent validation. **a**) Association of acute anti-spike antibody titers to PASC symptoms in Mount Sinai cohort. The y axis is the symptom assessed, and the x axis is the *P* value (-log10) for the association of the anti-spike antibody titers to the symptoms (linear mixed model, 2-sided t-test). The shape indicates the class of anti-spike antibody tested, and the color indicates whether the association is significant (BH FDR ≤ 0.05). **b**) Independent dataset validation of the non-association of acute anti-spike antibody titers to PASC. The x axis is the anti-spike antibody class and the y axis the titer measured for antibodies against the S1 domain of the spike protein during acute COVID-19. Each point represents a subject (n = 134 subjects, 85 with PASC). The color indicates the presence and absence of PASC and is defined in the legend. Two sided Mann-Whitney test unadjusted *P* values are shown between groups indicated by the brackets. Distributions are shown using box-and-whiskers plots (thick bar, median; box, 25th to 75th percentile, whiskers reach to the largest/smallest observations within 1.5 box-heights of the box.

In our hospitalized individuals, maximum COVID-19 severity^22^ and admission to the intensive care unit (ICU) were not significantly associated with symptoms (minimum severity ‘moderate’; Extended Data Fig. 1). Furthermore, demographics such as age and sex were not significantly correlated to PASC symptoms, with the sole exception of sex and hair loss (Extended Data Fig. 1). Among prior comorbidities, acute laboratory values and acute medications, there were eight significant associations with symptoms out of 2,780 tests (Supplementary Table 1c). These associations were not consistent across symptoms. Additionally, only sleep problems was significantly associated with acute anti-spike antibody titers (Extended Data Fig. 2a and Supplementary Table 1d). This general lack of association between acute anti-spike antibodies and PASC was validated in an independent dataset^17^, where neither anti-spike IgG nor IgA significantly associated with PASC (two-sided Mann–Whitney test, *P* ≥ 0.34; Extended Data Fig. 2b). Finally, significant co-occurrence between symptoms was observed with at least two distinct clusters related, respectively, to respiratory and neuropsychiatric traits (Fig. 2c and Extended Data Fig. 1).

### PASC symptoms associate with distinct CTS gene expression

**Extended Data Fig. 3:**
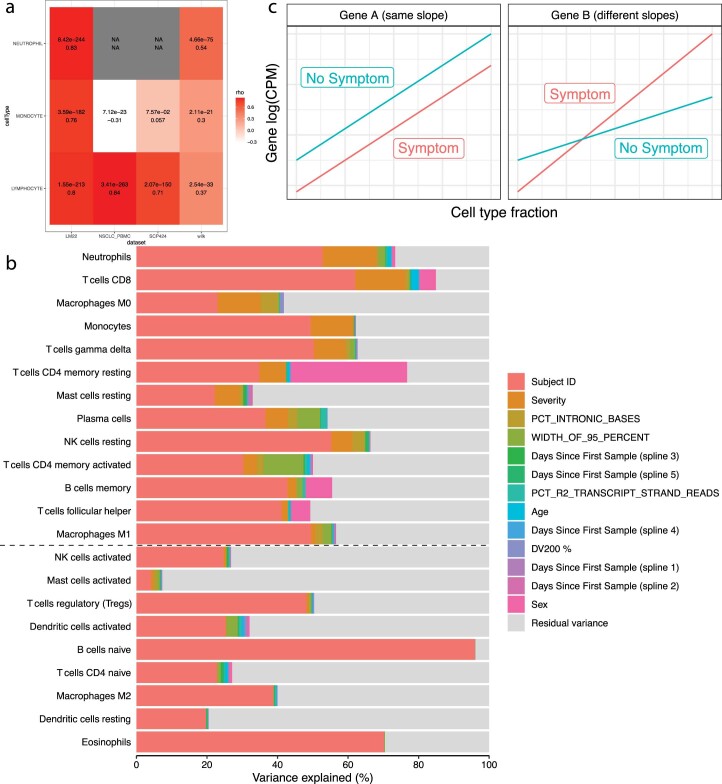
Cell-type fraction estimations and interaction model. **a**) Validation heatmap of estimated cell-type fractions with clinical complete blood counts. The x axis shows the literature reference dataset used for the deconvolution procedure and the y axis is the cell-type fractions validated. The colors represent the Pearson correlation (rho) values between the estimated cell-type fractions and the corresponding complete blood count from the clinical data. The correlation values and associated 2-sided *P* values adjusted for multiple testing (FWER, Holm’s method) and are noted in each box. Some reference data sets did not include neutrophils (indicated by gray boxes)**. b**) Estimated cell-type fraction variance explained by biological and technical variables. The x axis is the percent of variance of the cell-type fractions explained by covariates (colors) and the y axis the cell type assessed. Cell types are ordered by the decreasing percent of their variance explained by COVID-19 severity. The black dashed line represents the cutoff for inclusion in the cell-type-specific analyses. **c**) Schematic of interaction model for mock genes A and B. The x axis is the cell-type fraction of a specific cell-type of interest and the y axis the gene expression in log_2_(counts per million). The color represents the presence (red) and absence (blue) of a symptom. The left and right facets show a gene not differentially expressed (same slope) and a differentially expressed gene (different slopes) respectively.

We hypothesized that there is a relationship between the acute phase of COVID-19 and the development of post-acute sequelae that is detectable in blood gene expression. The RNA-seq of 361 acute blood samples from 165 individuals who had completed the PASC checklist was analyzed to identify acute gene expression patterns associating with symptoms 1 year after discharge. After thorough quality control of these data (Methods)^23–26^, cell type fractions were computationally estimated and validated using complete blood counts (Extended Data Fig. 3a)^27,28^. Higher acute plasma cell and lower follicular helper T cell fractions were associated with post-acute pneumonia and muscle pain, respectively, but most PASC symptoms had no significant associations with cell type fractions (Supplementary Table 1e). All gene expression traits were then tested for differential expression (DE) between the presence and absence of each symptom, accounting for ICU admission, COVID-19 severity at the time of blood sampling, sex, age and other confounding variables^29^. To identify genes differentially expressed within cells rather than genes whose differential abundance simply reflects cell type compositions, all analyses were performed while controlling for estimated cell type composition (Methods). No differentially expressed genes (DEGs) were found in whole blood for any symptoms.

**Fig. 3:**
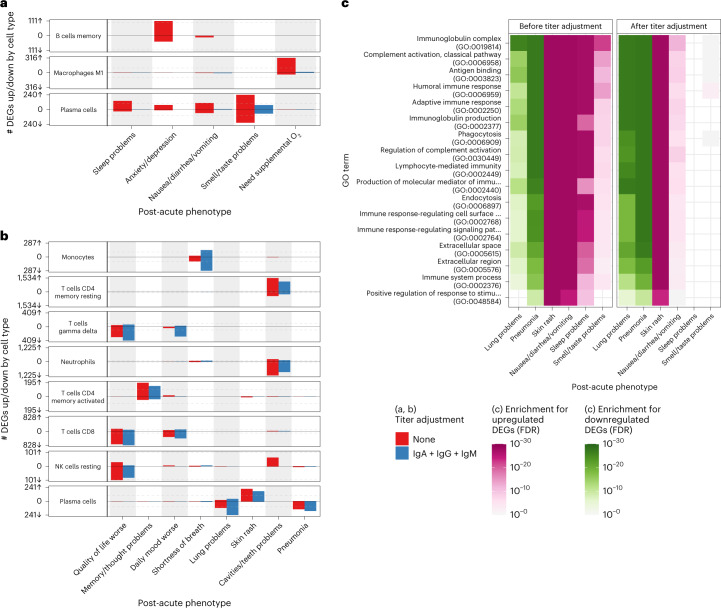
CTS DE for PASC symptoms. **a**,**b**, Anti-spike antibody titer-dependent (**a**) and titer-independent (**b**) CTS expression signatures. The *x* axes are PASC symptoms, and the *y* axes are the number of upregulated (up arrow) and downregulated (down arrow) DEGs at Benjamini–Hochberg FDR < 0.05. Symptoms are arranged in order of descending prevalence. Each facet presents DE results for the indicated cell type. The dashed gray lines provide a visual reference for the 100 DEG mark. The color of the bars indicates whether the signatures have been adjusted for anti-spike antibody titers. Only cell types and symptoms with more than 100 dependent/independent DEGs, respectively, are shown. **c**, GO term enrichments for plasma cell DEGs (one-sided Fisher’s exact tests, Benjamini–Hochberg adjustment for multiple testing). The *x* and *y* axes are the symptoms with more than 100 DEGs and GO terms, respectively. The union of the top three GO terms for all selected symptoms are shown. The color indicates the direction of the DEGs enriched for that term. Shading of color is representative of the FDR, and only FDRs < 0.05 are colored. The facets represent before (left) and after (right) controlling for anti-spike antibody titers. NK, natural killer.

**Extended Data Fig. 4:**
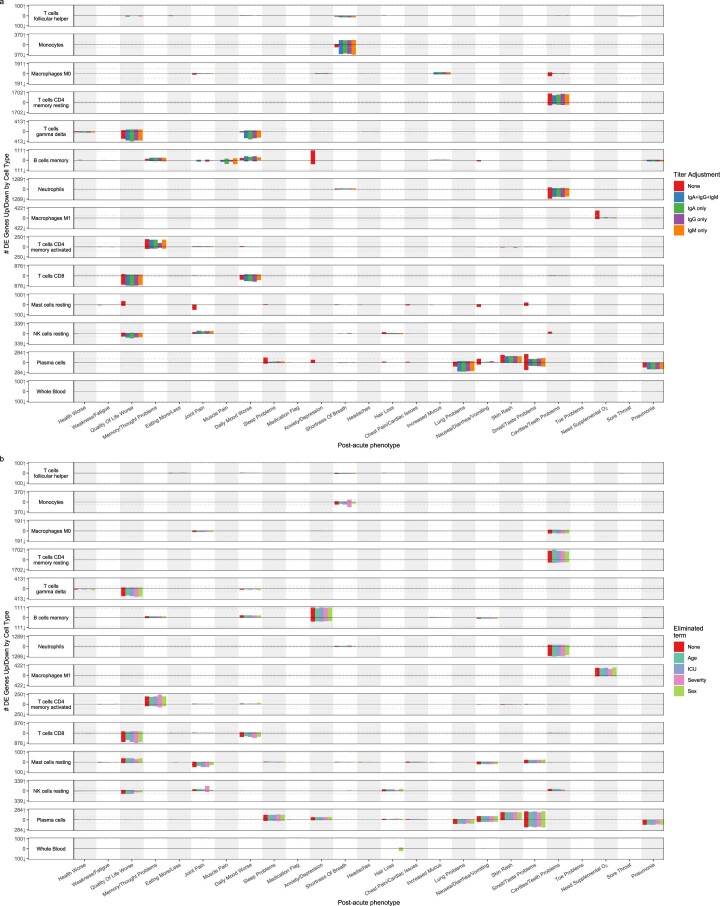
Cell-type-specific differential expression for PASC checklist items (full). The x axes are PASC checklist items (arranged in order of descending prevalence) and the y axes are the number of upregulated (above 0) and downregulated (below 0) DEGs at FDR≤0.05. Each row presents DE results for the indicated cell type. The dashed grey lines indicate the 100 DEG mark. The colors of the bars (defined in the legends) indicate (**a**) DE results for the specified anti-spike antibody titer adjustment (or no adjustment for titers, ‘None’), and (**b**) DE results when eliminating the specified term from the original model (shown as ‘None’). Note: ‘None’ and ‘IgA+IgG+IgM’ bars from Figure 3 are included here for ease of comparison.

**Extended Data Fig. 5:**
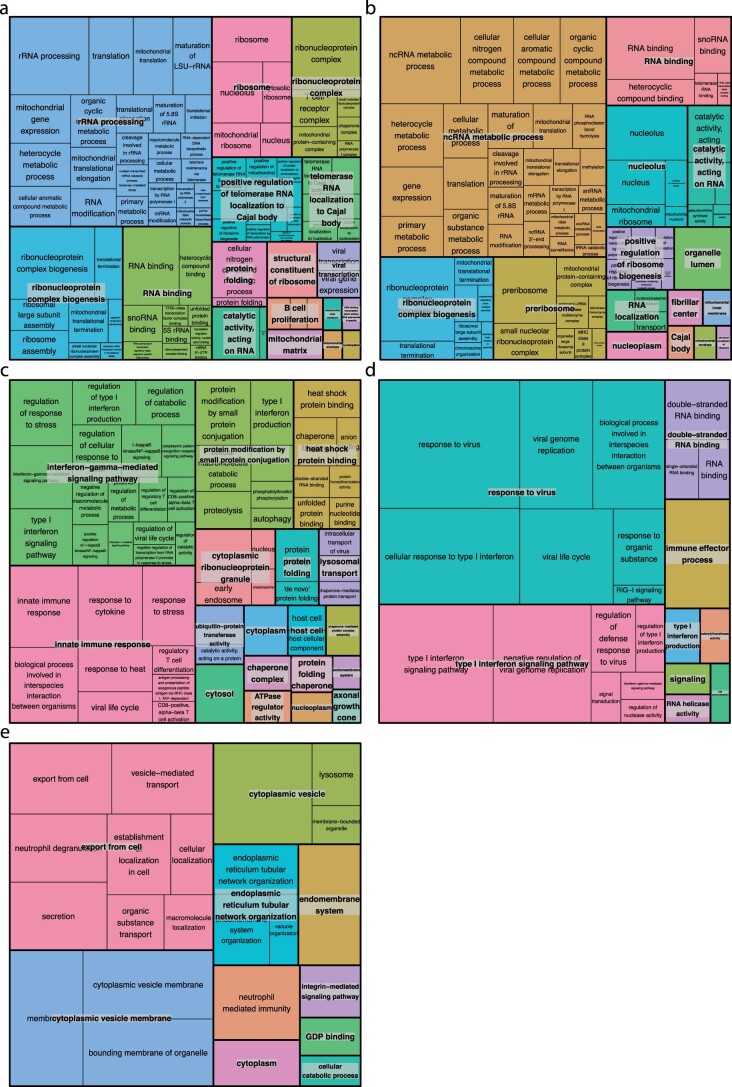
GO enrichments for DEGs for other PASC checklist items. Box sizes are relative to the -log_10_(adjusted *P* values) of the GO term enrichments for the corresponding DEGs and the term is noted in each box. Related terms are grouped by similarity and groupings are indicated by proximity and shared color. Consensus terms are indicated in bold for each group. **a**) Upregulated genes in memory resting CD4^+^ T cells for cavities/teeth problems. **b**) Downregulated genes in CD8^+^ T cells for quality of life. **c**) Upregulated genes in M1 macrophages for need supplemental O_2_. **d**) Upregulated genes in memory B cells for anxiety/depression. **e**) Upregulated genes in memory activated CD4^+^ T cells for memory/thought problems.

In addition, CTS differences in gene expression between presence and absence of symptoms were assessed using a DE model with an interaction term between the assessed symptom and a CTS estimated cell fraction (Fig. 1b and Methods). This model was fit for all cell types where at least 1% of the variation in estimated fractions was explained by COVID-19 severity (Extended Data Fig. 3b). For any given significant difference, CTS expression defined in this way encompasses the gene’s expression both inside and outside of that cell type but correlated to its relative fraction (Extended Data Fig. 3c). An extreme example of the latter would be a gene expressed only in cell type A that regulates the proliferation of cell type B. This CTS modeling approach was validated in an independent cohort of patients with COVID-19 with acute blood single-cell RNA-seq data that were assessed for different definitions of PASC at 2–3 months after onset of acute symptoms (Methods: ‘Validation of cell type interaction model in independent pseudo-bulk dataset’)^16^. Many symptoms showed significant DE in CTS tests (Fig. 3a,b; Extended Data Fig. 4a, red bars; and Supplementary Table 2a), and their respective signatures were further annotated for known biology using Gene Ontology (GO) term enrichment analysis (Fig. 3c, Supplementary Table 3a–l and Extended Data Fig. 5)^30^. The detection of the CTS DEGs was robust to covariate selection (Methods: ‘Differential expression analyses’; Extended Data Fig. 4b; and Supplementary Table 4a). To verify that our models captured CTS DE, DEGs were compared to the corresponding CTS markers from the literature, and a significant overlap was found in most instances (Supplementary Table 5a). The results presented below focus primarily on cell types whose markers were enriched in the DEGs found for those cell types. Genes with significantly higher and lower expression in patients with a symptom are hereafter referred to as upregulated and downregulated, respectively. Plasma cells had at least 100 DEGs for the largest number of symptoms: sleep problems, lung problems, nausea/diarrhea/vomiting, skin rash, smell/taste problems and pneumonia (Fig. 3a,b). Notably, the DEGs for pneumonia were almost entirely downregulated (Fig. 3b), not simply recapitulating the association between pneumonia and higher plasma cell fraction described above (Supplementary Table 1e). Of other cell types and symptoms with more than 100 DEGs (Fig. 3a,b), CD8^+^ and γδ T cells were associated with a worse quality of life; memory resting CD4^+^ T cells and neutrophils were associated with cavities/teeth problems; and memory-activated CD4^+^ T cells were associated with memory/thought problems.

### PASC symptom DE signatures suggest multiple etiologies

**Fig. 4:**
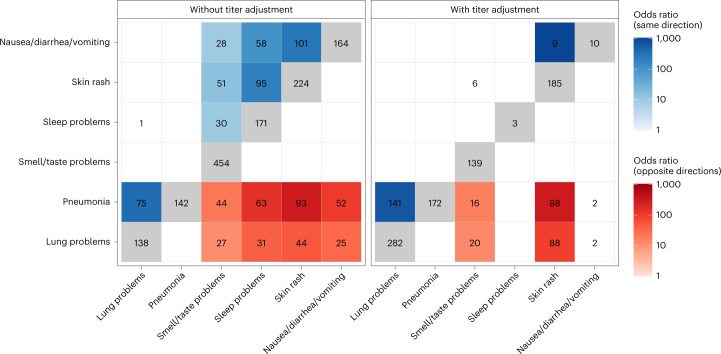
Shared plasma cell DEGs between PASC symptoms. The *x* and *y* axes are the PASC symptoms associated with more than 100 DEGs. The numbers in each box are the numbers of shared DEGs between the two symptoms defined in the axes, and the color and position represent whether they are same-direction (blue, upper left), opposite-direction (red, lower right) or the total number of DEGs for that checklist item (gray, diagonal). The shadings of red and blue are the ORs of the one-sided Fisher’s exact tests for the enrichment of overlapping genes in that box and are shown only if the associated enrichment adjusted *P* < 0.05 (FWER, Holm’s method). The left and right facets represent the shared DEGs before and after adjustment for anti-spike antibody titers, respectively. Symptoms in rows and columns are ordered by hierarchical clustering and optimal leaf ordering based on the shared same-direction DEGs. Source data

**Extended Data Fig. 6:**
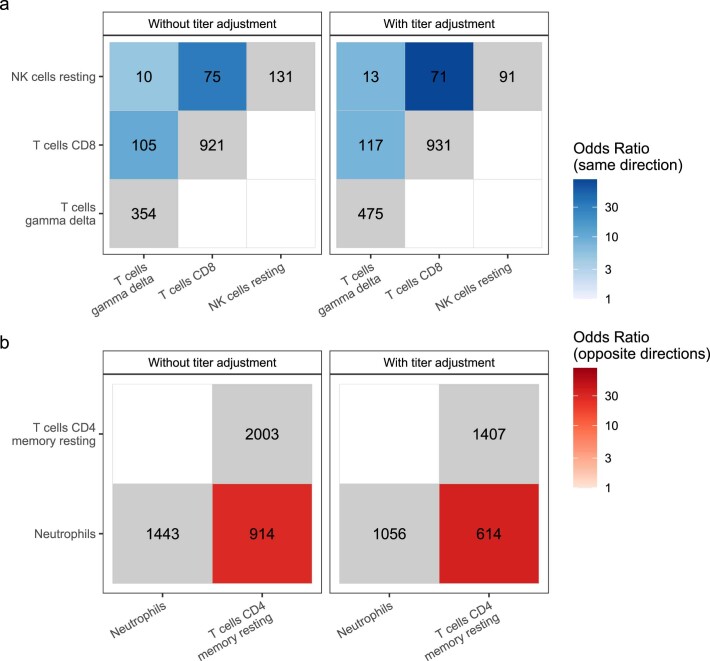
Shared PASC checklist DEGs between cell-types. The x and y axes are the cell types associated with more than 100 DEGs. The numbers in each box are the numbers of shared DEGs between the two checklist items defined in the axes, and the color represents whether they are same-direction (blue), opposite direction (red) or the total number of DEGs for that checklist item (grey). The shadings of red and blue are the ORs of the 1-sided Fisher’s exact tests for the enrichment of shared DEGs in that box, and are shown only if the associated enrichment adjusted *P* value < 0.05 (FWER, Holm’s method). The left and right facets represent the shared DEGs before and after adjustment for anti-spike antibody titers respectively. Symptoms in rows and columns are ordered by hierarchical clustering and optimal leaf ordering based on the shared same-direction DEGs. **a**) Quality of life shared DEGs. **b**) Cavities and teeth problems shared DEGs. Source data

To define the common molecular architectures of acute mechanisms leading to different PASC symptoms, we examined how CTS DE signatures were shared between symptoms and cell types (Fig. 4 and Extended Data Fig. 6). When comparing DE signatures, we define ‘opposite-direction DEGs’ as the genes upregulated in one signature and downregulated in the other; likewise, ‘same-direction DEGs’ are genes either upregulated in both signatures or downregulated in both signatures. Pairwise comparison of symptoms for same-direction DEGs in plasma cells revealed two symptom clusters, implying multiple etiologies for different PASC symptoms (Fig. 4). Lung problems and pneumonia formed one (‘plasma cell pulmonary cluster’), and sleep problems, nausea/diarrhea/vomiting, skin rash and smell/taste problems formed the other (‘plasma cell miscellaneous cluster’). Notably, immunoglobulin-related GO terms were downregulated in the plasma cell pulmonary cluster and upregulated in the miscellaneous cluster (Fig. 3c). Additionally, when symptoms between plasma cell clusters were compared, significant enrichment was observed only for opposite-direction DEGs (Fig. 4). This observation, consistent with the infrequent co-occurrence of symptoms in different clusters, emphasizes the clinical relevance of these molecularly defined clusters (Fig. 2c).

For quality of life, same-direction DEGs were significantly enriched between CD8^+^ and γδ T cells (Extended Data Fig. 6a). Memory resting CD4^+^ T cells and neutrophils had a significant enrichment of opposite-direction DEGs for cavities/teeth problems (Extended Data Fig. 6b). Both CD4^+^ T cell and neutrophil DEGs were enriched for CD4^+^ T cell marker genes (CD4^+^ T cell DEG enrichment: odds ratio (OR) = 3.5, *P* = 2.2 × 10^−22^; neutrophil DEG enrichment: OR = 2.5, *P* = 1.04 × 10^−8^), whereas neither was enriched for neutrophil marker genes (*P* > 0.05). This asymmetry suggests that the neutrophil-specific interaction model is identifying CD4^+^ T cell DEGs, likely due to the negative correlation between the estimated fractions of these cell types (Pearson correlation = −0.60, *P* = 3.12 × 10^−137^).

### DE signatures confirm multiple etiologies for PASC symptoms

**Extended Data Fig. 7:**
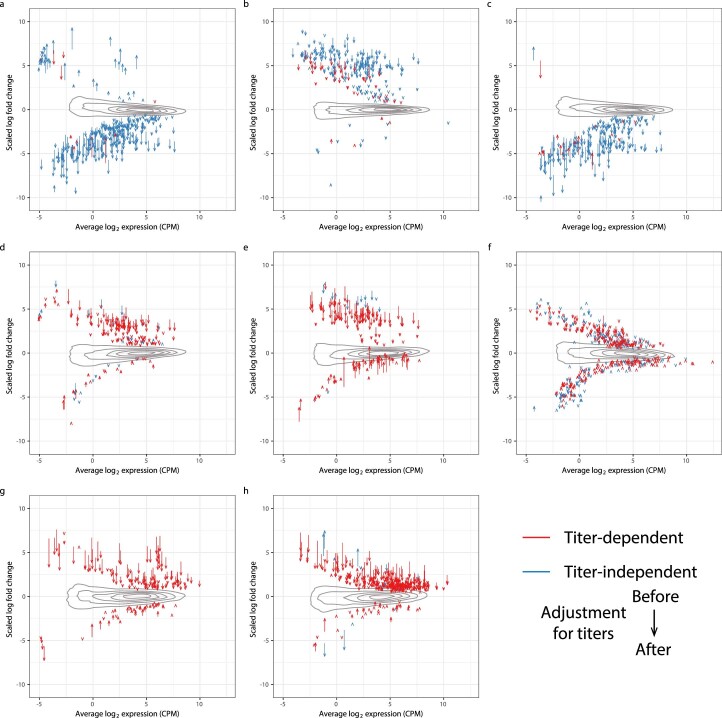
Delta-MA plot of anti-spike antibody titer effect on differential expression log (fold change). The x and y axes represent the average normalized gene expression and the differential expression log fold change (logFC) respectively. Each arrow shows a single DEG. The arrow colors indicate the anti-spike antibody titer dependent (red) and independent (blue) DEGs. The contours show the distribution of all logFC values before controlling for antibody titers. The effect of controlling for antibody titers on DEGs is shown by the arrows, with the arrow tail being the logFC before adjustment and the arrow head the logFC after adjustment. LogFC values in each panel are scaled such that the root mean square logFC before adjustment is equal to 1. **a**) Lung problems in plasma cells. **b)** Skin rash in plasma cells. c) Pneumonia in plasma cells. **d**) Sleep problems in plasma cells. **e**) Nausea/diarrhea/vomiting in plasma cells. **f**) Smell/taste problems in plasma cells. **g**) Anxiety/depression in memory B cells. **h**) Need Supplemental O_2_ in M1 macrophages.

Given the many symptoms associated with more than 100 DEGs in plasma cells, whose primary function is to produce antibodies, we assessed whether CTS DEGs were dependent on the antibody response to the SARS-CoV-2 spike protein. To identify DEGs that are independent of the anti-spike antibody titers^31^, all gene expression analyses were repeated while controlling for blood sample titers of anti-spike IgG, IgA and IgM (Figs. 1c and 3; Extended Data Fig. 4a, blue bars; and Supplementary Tables 2b, 3m–v and 5b). Compared to the DEGs identified above, those that are no longer significant after controlling for titers are defined as titer-dependent, whereas those remaining significant are titer-independent. This computational inference of titer-dependence was validated by stratifying samples by anti-spike antibody titers into low-titer and high-titer strata, fitting the same DE model to each stratum and comparing the full dataset DE results to the expression patterns in the strata (Methods: ‘Titer-stratified differential expression models’). Adjusting for titers resulted in a near-complete attenuation of both the magnitude and significance of the plasma cell DEG signal for a subset of the plasma cell miscellaneous cluster (sleep problems, nausea/diarrhea/vomiting and smell/taste problems), establishing these as titer-dependent, thereby demonstrating an explicit link between this subset of symptoms and the host response to SARS-CoV-2 infection (Fig. 3 and Extended Data Fig. 7a–c). This titer-dependency, also observed when controlling for titers of any single class, is not attributable to any specific class of anti-spike antibody (Extended Data Fig. 4a, blue, green, purple and orange bars; and Supplementary Table 4b). For two of these symptoms (nausea/diarrhea/vomiting and sleep problems), the upregulation of immunoglobulin-related GO terms was likewise absent when controlling for antibody titers (Fig. 3c). In contrast, skin rash and the plasma cell pulmonary cluster symptoms showed little to no attenuation of the plasma cell DEGs and similar GO term enrichments, establishing these as titer-independent (Fig. 3b,c and Extended Data Fig. 7d–f). These dependence patterns on anti-spike antibody titers confirm the presence of at least two distinct etiologies for the plasma cell pulmonary and miscellaneous clusters. Two additional signatures were largely titer-dependent: memory B cells with anxiety/depression and M1 macrophages with the need for supplemental oxygen (Fig. 3 and Extended Data Fig. 7g,h). Similarly to DEGs described in the previous sections, DEGs identified after controlling for anti-spike antibody titers were mostly enriched for the corresponding cell type marker genes from the literature (Supplementary Table 5b).

Patterns of shared DEG signatures across cell types and symptoms were re-computed after controlling for anti-spike antibody titers. Same-direction DEG patterns were generally conserved among titer-independent signatures (Fig. 4 and Extended Data Fig. 6a). Notably, the plasma cell miscellaneous cluster was divided into two components: one entirely titer-dependent (sleep problems and nausea/diarrhea/vomiting) and one partially titer-dependent (skin rash and smell/taste problems). In particular, DEGs shared between skin rash and smell/taste problems were primarily titer-dependent, whereas DEGs unique to each symptom were largely titer-independent (Fig. 4). Furthermore, both symptoms retained their opposite-direction DEGs with the symptoms in the titer-independent plasma cell pulmonary cluster. These observations suggest additional etiological divergence within the plasma cell miscellaneous cluster.

### Validation of titer-independent immunoglobulin DEGs

**Fig. 5:**
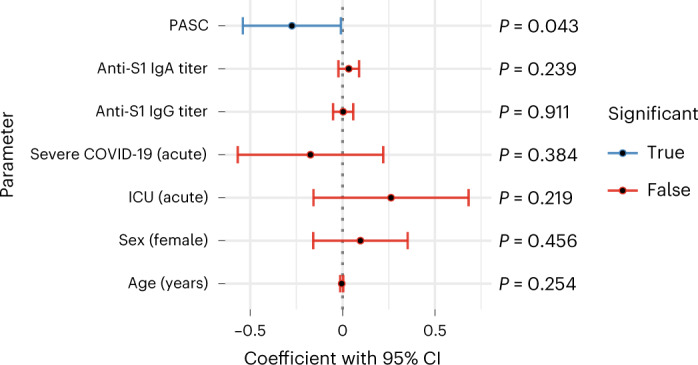
Independent dataset validation of lower antibody production in PASC. Plot of linear model coefficients and *P* values (two-sided *t*-test, d.f. = 126, no adjustment for multiple testing) for prediction of the product of total IgM and total IgG3 (*n* = 134 individuals, 85 with PASC). The *y* axis lists all non-intercept coefficients, including presence of PASC, titers of antibodies against the S1 domain of the spike protein, severity, ICU admission, sex and age. The *x* axis shows the coefficient values, with the black center point showing the fitted value and the error bars showing the 95% confidence interval (CI) about this value. CIs that include 0 are colored red, whereas those that indicate a significant difference from 0 (*P* < 0.05) are colored blue.

**Extended Data Fig. 8:**
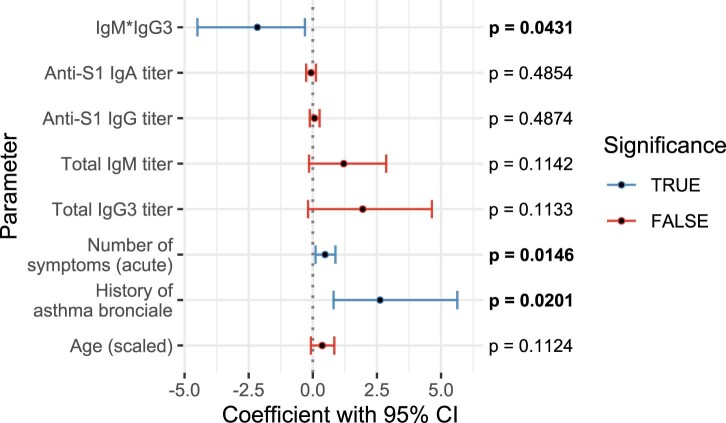
PASC prediction by total Ig in independent data set is independent of anti-spike Ig. Plot of logistic regression model and *P* values (2-sided likelihood ratio test, no adjustment for multiple testing) for prediction of PASC (n = 134 subjects, 85 with PASC). The y axis lists all non-intercept coefficients, and the x axis shows the coefficient values, with the black center point showing the fitted value and the error bars showing the 95% confidence interval (CI) about this value. CIs that include 0 are colored red, while those that indicate a significant difference from 0 (p < 0.05) are colored blue.

As described above, the plasma cell pulmonary cluster symptoms showed no association with anti-spike antibody titers, and, although plasma cell DEGs for these symptoms were titer-independent and largely downregulated, they were nevertheless enriched for GO terms related to immunoglobulin production and function (Fig. 3b,c and Supplementary Tables 1d and 3h,r). Given that antibodies specific to an active pathogen comprise only a small fraction of total immunoglobulin^32–34^, these seemingly contradictory results could be explained by variations in total immunoglobulin that are unrelated to levels of anti-spike immunoglobulin. To test this hypothesis, we leveraged an independent dataset where both anti-spike and total antibody titers were measured during acute COVID-19 in individuals later assessed for PASC^17^. There, the authors show that a lower acute titer of either total IgM or total IgG3 is predictive of subsequent PASC development^17^. We confirmed that this predictive value of total IgG3 and IgM held when controlling for titers of anti-spike IgA and IgG (Extended Data Fig. 8). Because this predictive value of total immunoglobulin titer to PASC does not necessarily imply the reverse, we validated that the interaction of acute total IgG3 and IgM titers was significantly lower in individuals who later developed PASC. Again, this result held while controlling for titers of anti-spike IgA and IgG, demonstrating that this association is truly independent of the anti-spike-specific antibody response (Fig. 5). These results emphasize that the downregulation of immunoglobulin genes in the plasma cell pulmonary cluster can be explained by more than just the anti-spike-specific antibody production.

## Discussion

The long-term health consequences after SARS-CoV-2 infection, described collectively as PASC, are recognized to have a major negative impact on human health^20^. This report presents a large-scale transcriptome-wide investigation of blood gene expression changes occurring during acute COVID-19 that associate with subsequent PASC. With a cohort of this size, composed exclusively of hospitalized individuals with long-term (>6 months) follow-up, this study is uniquely powered to begin characterizing the molecular aspects of the acute host response to SARS-CoV-2 infection that eventually develop into PASC. Multiple CTS acute gene expression patterns that associate with individual PASC symptoms are identified, suggesting distinct etiologies for different subsets of symptoms. These expression patterns, which define clusters of symptoms based not simply on symptom co-occurrence but also on shared gene expression patterns, establish acute COVID-19 as a critical early window in the pathogenesis of PASC that should be captured in future study designs. Plasma cells are identified as important to the etiology of PASC, with over 100 DEGs in six symptoms, defining two distinct clusters. The plasma cell pulmonary cluster was mostly associated with lower expression of genes involved in antibody production and function, whereas the plasma cell miscellaneous cluster was associated with higher expression of many of those same genes (Figs. 3 and 4). The opposing gene expression patterns observed between these clusters, and the varying dependency of these plasma cell DEGs on anti-spike antibody titers, show at least two etiologies of PASC symptoms, already detectable and molecularly distinct during acute COVID-19. The existence of these distinct etiologies provides a plausible explanation for the lack of observed co-occurrence of symptoms across clusters.

The higher expression of genes involved in antibody production and function in the plasma cell miscellaneous symptoms cluster largely depends on the anti-spike antibody response, explicitly linking these symptoms to the host immune response to the virus. Computational analyses have identified SARS-CoV-2 antigens exhibiting structural similarities to human antigens, a phenomenon known as molecular mimicry^35^. Additional studies find autoreactivity in SARS-CoV-2-specific antibodies, such as monoclonal antibodies against the SARS-CoV-2 spike and nucleocapsid proteins that reacted against human antigens^36^, and monoclonal antibodies derived from a patient with COVID-19 binding to both SARS-CoV-2 antigens and human naive B cells^37^. Cross-reactivity with SARS-CoV-2 and human antigens, thus, possibly explains the anti-spike-dependent gene expression patterns observed here for the plasma cell miscellaneous cluster. Alternatively, dependence on the host response to SARS-CoV-2 infection could simply represent a generalized immune system dysfunction. For example, SARS-CoV-2 infection is reported to induce a relaxation of peripheral tolerance in B cells, allowing the emergence of autoreactive antibodies linked to autoimmune disorders^37^. Furthermore, persistence of autoreactivity after acute COVID-19 is shown to associate with PASC^19^. Notably, two symptoms in this plasma cell miscellaneous cluster (skin rash and smell/taste problems) also had separate titer-independent DE signatures, suggesting additional divergent etiologies.

The downregulation observed in the plasma cell pulmonary cluster was independent of the anti-spike antibody titers, suggesting a non-specific downregulation of humoral immune activity underlying pulmonary symptoms. This is supported by the observation in an independent dataset of a lower product of titers of acute total IgG3 and IgM independently of anti-spike antibody titers in individuals who later developed PASC (Fig. 5). The lower expression of immunoglobulin-related genes in the plasma cell pulmonary cluster is consistent with reported associations between deficiencies in antibody production and recurrent pulmonary disease^38–41^. This observed downregulation possibly represents a pre-existing antibody deficiency that, in combination with COVID-19, may result in persistent pulmonary symptoms. Autoantibodies, documented in COVID-19 (refs. ^20,42^), could also explain the independence of this signal from anti-spike antibody titers. Indeed, post-acute cough and sputum were shown to be associated with higher levels of anti-IFN-a2 and anti-U1-snRNP, both during and after acute COVID-19 (ref. ^16^). This reported association with higher autoantibody titers is consistent with the results presented here, given the significant post-acute association of autoantibodies with lower total IgM and higher total IgG1 and the lack of significant association of both acute and post-acute IgG1 titer with PASC^17,43^. Hence, the distinct etiologies identified for the plasma cell pulmonary and miscellaneous clusters independently corroborate current knowledge of the aftermath of SARS-CoV-2 infection.

Beyond plasma cells, gene expression in other cell types was also associated with PASC symptoms (Fig. 3a,b), implying potential additional etiologies. With the reported importance of many of these cell type/symptom combinations to acute COVID-19, their CTS signature associations with PASC symptoms could represent a lack of resolution of acute COVID-19 processes as well as molecular events triggering cascades that develop into PASC. Monocytes with distinct gene expression patterns were shown to be present in the blood and to infiltrate the lungs during acute COVID-19 (refs. ^44,45^), potentially representing the acute processes in monocytes that lead to shortness of breath (Fig. 3b). Similarly, macrophage gene expression profiles were implicated in tissue damage caused by inflammation in lungs infected by SARS-CoV-2 (ref. ^46^), possibly related to DEGs associated with need for supplemental oxygen (Fig. 3a). The connection between acute gene expression in CD8^+^ T cells and self-assessed quality of life long term (Fig. 3b), together with the known association of SARS-CoV-2-specific CD8^+^ T cell responses with acute COVID-19 severity^47–49^, also warrants further study. Other cell type/symptom combinations, not directly implicated in acute COVID-19 but consistent with known dysregulation of CTS processes in disorders with similar clinical manifestation, need to be further studied to understand their contribution to PASC. Memory CD4^+^ T cells, whose gene expression is associated with memory/thought problems (Fig. 3b), are known to be active in the brain^50^ and have been shown in mouse models to play a functional role in memory^51^. Finally, the detection of DEGs in CD4^+^ T cells with cavities/teeth problems (Fig. 3b) is compatible with the known association of expression of the CD4 gene in T cells with the occurrence of early childhood caries^52^. More generally, the complex pattern of associations seen in the CTS DEGs for multiple innate and adaptive immune cell types further supports the hypothesis that PASC is a complex set of traits with multiple etiologies beyond the two identified in plasma cells.

Although this study brings forth a robust initial characterization of the processes that occur during acute COVID-19 that associate with PASC, limitations remain, along with opportunities for future studies. First, PASC symptoms were not clinically evaluated but, rather, self-assessed, and data may be confounded by individuals’ choice to complete the checklist. Furthermore, with PASC still poorly defined, our checklist represents an adequate attempt to capture the full breadth of symptoms experienced after COVID-19, but a precise definition of relevant phenotypes and subtypes would increase power to detect meaningful signal in future studies. Although PASC is known to occur after mild cases of COVID-19 not requiring hospitalization and asymptomatic SARS-CoV-2 infections^6,53^, the Mount Sinai cohort is composed exclusively of hospitalized patients with COVID-19, limiting the conclusions to that population. Future works will need to replicate and further characterize the relationship between acute COVID-19 and PASC both within and outside of the hospitalized setting, to elucidate the mechanisms leading to PASC and confirm its causal relationship with SARS-CoV-2 infection. In addition, knowing patients’ true infection dates would allow us to control for the timing of blood sampling with respect to acute disease course, likely augmenting our findings, especially for cell types that may appear in circulation only transiently during infection, such as plasma cells^54^. Furthermore, we used, from among several potentially valid analysis strategies, an interaction model that we showed to effectively capture CTS DEGs. The usefulness of this modeling approach requires further development and evaluation to identify the best strategy for identifying CTS DEGs in bulk RNA-seq data. Additionally, future works can use the RNA-seq data generated here to explore the breadth of the adaptive immune repertoire^55,56^. The dataset presented here does not include post-acute molecular data, preventing us from directly characterizing the connection between acute molecular signals and the molecular components of PASC. Ongoing efforts to understand the etiologies of PASC will require complete molecular characterization of both acute and post-acute phases in the same individuals. Lastly, although we report many CTS DEGs for several PASC symptoms, the analysis is underpowered for some combinations of cell types and symptoms, so the DEGs identified are likely only a subset of the true acute phase expression signatures that will be discovered by studying larger cohorts in the future.

In conclusion, at least two divergent etiologies were identified for different sets of PASC symptoms, one dependent and one independent from the antibody response to the SARS-CoV-2 spike protein. The discovery of the association of gene expression during acute COVID-19 with PASC symptoms 1 year after discharge establishes the existence of direct connections between the acute and post-acute phases. Although designing studies to capture patients during both acute COVID-19 and the post-acute phase undoubtedly entails considerable challenges, the work presented here demonstrates the need to consider the acute phase to better understand the development of long-term symptoms. Furthermore, with such designs, predictive molecular biomarkers of specific PASC symptoms could be identified. By controlling for the clinical presentation of COVID-19, the analyses presented here also demonstrate that the molecular processes leading to PASC are not explained simply by acute severity. Although additional studies will be required to determine if our findings generalize to mild COVID-19 and asymptomatic infections, this lack of dependence on disease severity is consistent with the reported occurrence of PASC across the range of severity for SARS-CoV-2 infection^1,2,57–59^. It is also anticipated that future studies of the relationship between acute infection and PASC will define additional symptom clusters with common underlying mechanisms. Finally, knowledge of symptom-specific mechanisms will present opportunities to investigate precision treatment and prevention strategies.

## Methods

### Ethics statement

This study was approved by the Human Research Protection Program at the Icahn School of Medicine at Mount Sinai (STUDY-20-00341). All patients admitted to the Mount Sinai Health System were made aware of the research study by a notice included in their hospital intake packet. The notice outlined details of the specimen collection and planned research, and it provided instructions on how to opt out of the study. Flyers announcing the study were also posted in the hospital, and a video was run on the in-room hospital video channel. Given the hurdles of consenting acute patients in isolation rooms, the Human Research Protection Program allowed for sample collection, which occurred at the time of clinical collection, before obtaining research consent. Limited existing clinical data obtained from the medical record were collected and associated with the samples. As the research laboratory processing needed to begin proximal to sample collection, a portion of the data was generated before obtaining informed consent. During or after hospitalization, research participants and/or their legally authorized representative provided consent to the research study, including genetic profiling for research and data sharing on an individual level. In those circumstances where consent could not be obtained (13.8% of individuals, 0% of individuals who completed the post-discharge checklist), data already generated could continue to be used for analysis purposes only when not doing so would have compromised the scientific integrity of the work. In this study of PASC, data from withdrawn and unconsented individuals were used only for quality control. The data were not identifiable to the researchers doing the analyses.

### Sample collection

Patients presenting to the Mount Sinai Health System between April and June 2020 were enrolled through daily manual review of new hospitalizations for COVID-19. Patients did not receive compensation for their participation in the study. Blood collection was performed in conjunction with routine clinical blood draws throughout participants’ hospital stays. Research specimens were brought to Biosafety Level 2-plus facilities for accessioning, processing and storage of serum, plasma, whole blood and peripheral blood mononuclear cells (PBMCs). The whole blood used for RNA-seq was collected in Tempus RNA Blood Tubes (Thermo Fisher Scientific, 4342792). As soon as possible after blood collection, tubes were shaken and stored at −80 °C. Blood used for Olink and ELISA were collected in SST tubes (Becton Dickinson, 367985), and blood used for whole-genome sequencing (WGS) was collected in CPT Vacutainer tubes (Becton Dickinson, 362761). Blood in SST tubes was centrifuged to extract serum, aliquoted and stored at −80 °C in cryovials (Crystalgen, 19335-6SPR). Blood from CPT tubes was aliquoted for WGS and stored at −80 °C in cryovials (Crystalgen, 19335-6SPR).

### ELISA

Sera were evaluated by ELISA for IgG, IgA or IgM antibody to the full-length spike protein (original variant), using methods previously described^31^. In brief, 96-well half-area cluster plates (Corning Costar) were coated with 30 μl of *Escherichia coli*-produced recombinant spike protein (gift from N. Herrera and S. Almo, Albert Einstein College of Medicine) diluted in carbonate/bicarbonate buffer (pH 9.4; Sigma-Aldrich, C3041-100CAP) at 1 μg ml^−1^. After incubation overnight at 4 °C, plates were washed with PBS with 0.1% Tween 20 (TPBS) and then blocked for 1 hour with 5% nonfat dry milk in TPBS (blocking buffer). Beginning at 1:100, four-fold serial dilutions of participant and control sera were prepared in blocking buffer and added to individual wells. After 2 hours at room temperature and washing, secondary antibody (goat anti-human IgG, IgA or IgM AP conjugate; SouthernBiotech, 2040-04, 2050-04 and 2020-04, respectively; diluted 1/4,500, 1/4,000 and 1/3,000, respectively) were added to all wells, incubated for 1 hour at room temperature and then washed. Attophos substrate (Promega, S1001/S1000) was added to each well for 30 minutes. 3N NaOH was added to stop the reaction, and fluorescence was recorded on a Biotek Synergy fluorescent plate reader. The FORECAST function in Microsoft Excel was used to calculate the antibody titer of participants’ sera, which was defined as the reciprocal of the serum dilution that yields a fluorescent intensity ten times greater than the cutoff value. The cutoff value was defined as the average fluorescence obtained from the first four dilutions of serially diluted normal donor serum pool (negative control). Antibody titers ≥100 were considered positive. Accuracy was established in comparison to a CLIA-approved assay for the spike receptor binding domain^31^ with specificity >0.96 and higher sensitivity in the low titer range.

### Olink data generation

Serum samples were analyzed for a panel of 92 circulating proteins associated with human inflammatory conditions using the Olink multiplex assay (Olink Target 96 Inflammation, Olink Bioscience) according to the manufacturer’s instructions. Incubation master mix containing pairs of oligonucleotide-labeled antibodies to each protein was added to the samples and incubated for 16 hours at 4 °C. Each protein was targeted with two different epitope-specific antibodies to increase the specificity of the assay. Presence of target protein in samples brings partner probes into close proximity to each other, allowing formation of a double-stranded oligonucleotide polymerase chain reaction (PCR) target. The next day, extension master mix was added to samples to cause specific target sequences to be detected and generate amplicons using PCR in a 96-well plate. For detection of specific protein, a Dynamic Array integrated fluidic circuit 96 × 96 chip was primed, loaded with 92 protein-specific primers and mixed with sample amplicons, including three inter-plate controls and three negative controls. Real-time microfluidic quantitative PCR was performed in Biomark (Fluidigm) for target protein quantification. Data were analyzed using real-time PCR analysis software via the ΔΔCt method and NPX (Normalized Protein Expression) Manager. Data were normalized using internal controls in each sample, inter-plate controls to normalize across plates and a correction factor calculated by Olink from negative controls, producing NPX values proportional to the log_2_ of the protein concentration.

### Clinical data generation

Clinical data elements (CDEs) were ascertained by employing a three-pronged strategy. First, automated extraction of structured CDEs from electronic health records (EHRs) was employed, constructing an exhaustive database of over 1,000 CDEs that included demographics, vitals, comorbidities, clinical laboratory test results and medications. For CDEs with multiple entries in a 24-hour window, entries were collapsed by calculating the median for numerical CDEs and retaining the most severe entry for categorical CDEs. Groups of CDEs were combined to derive additional CDEs, such as 24-hour summaries of COVID-19 severity (defined as four categories: control (that is, SARS-CoV-2 negative), moderate, severe and severe with end organ damage (EOD))^22^. Second, we employed manual chart review by subject matter experts to extract unstructured CDEs from free text of clinical notes, such as dates of COVID-19 symptoms onset. For these two components of our CDE ascertainment strategy, manual and automated quality control checks were performed to verify consistency and correctness of the data. The third approach to CDE ascertainment was a checklist of health changes after COVID-19 (or, for controls, after hospitalization). The checklist was constructed when anecdotal reports of post-acute sequelae were just beginning to surface, with the goal of capturing a set of CDEs that collectively reflected the prevailing view of PASC at the time (Supplementary Table 1a,b). Checklists were completed from February through July 2021 by study participants remotely via a webform after the acute COVID-19 hospitalization, with a clinical research coordinator on the phone for assistance. Checklist items were generally of two types: those that assessed clinical deterioration (for example, quality of life worsening since COVID-19, ‘general’) and those that assessed symptom presence (for example, the emergence of memory issues since COVID-19, ‘symptoms’). All were coded as Boolean variables, with ‘true’ indicating either clinical deterioration or the presence of symptom. We removed symptoms reported by *n* ≤ 5 individuals with RNA-seq from further analyses to allow models to converge. For the small number of participants that completed the checklist on more than one post-acute timepoint, answers were collapsed into a single value, with a ‘true’ value for any item coded as such at any of the timepoints. All individuals without any survey answers were assigned to a third ‘unknown’ group for each item.

### DNA extraction

After blood collection, tubes were shaken, and blood was aliquoted into cryovials and stored at −80 °C. The MagMax DNA Multi-Sample Ultra 2.0 Kit protocol (Thermo Fisher Scientific, A36570) was used to isolate DNA from 0.2 ml of blood, following the manufacturer’s instructions. A KingFisher Flex machine was used to automate the isolation of 96 samples at once with the MagMAX_Ultra2_200μL_FLEX program. In brief, frozen blood cryovials were thawed at room temperature before DNA extraction. Next, processing plates were labeled and assembled. Plate 1 contained 500 μl of Wash 1 solution; plate 2 contained 500 μl of Wash 2 solution; plate 3 contained 500 μl of Wash 2 solution; and plate 4 contained 75 μl of elution buffer. The sample plate was then prepared by first adding 20 μl of enhancer solution and then 200 μl of the blood sample and 20 μl of proteinase K, in that order. All plates were then put into the KingFisher to start DNA extraction. In the middle of the program, 220 μl of DNA Binding Bead Mix was added to each sample well. At the end of the run, the elution plate was removed from the instrument, and DNA samples were transferred to a skirted 96-well plate. If there were excess beads in the DNA samples, the beads were collected on a plate magnet, and purified DNA samples were transferred into a new skirted 96-well plate.

### WGS

Once isolated DNA passed quality control, we conducted WGS library preparation with the Nextera DNA Flex Library Preparation Kit (Illumina, 20018705), using 250–500 ng of genomic DNA as input and by following the manufacturer’s protocol. In brief, genomic DNA samples were simultaneously fragmented and ligated with adapters by tagmentation. Tagmented DNA fragments were then amplified using a limited number of PCR cycles to ligate indexes to each template. Quality of final libraries was then validated on the Agilent TapeStation 4200 using High Sensitivity D1000 screen tape (Agilent Technologies, G2991AA). Each library’s concentration was measured on a Quant-iT High Sensitivity dsDNA Assay Kit (Thermo Fisher Scientific, Q33120). After library preparation, we performed WGS targeting 30× coverage using Illumina paired-end, short-read sequencing technology on the NovaSeq 6000. To achieve even coverage across patient genomes, these libraries were sequenced at a multiplex of 24–29 samples per batch and assigned to the S4 NovaSeq flow cell to account for batch size. This configuration enabled 150-bp paired-end reads into resulting FASTQ files in 2–5 days per batch that were sent through primary data quality control using MultiQC to assess read depth and quality metrics.

### Selection of RNA-seq batch controls

Technical effects emerging from batching of samples at various processing steps in gene expression studies (for example, extraction and sequencing) are a large confounding variable in downstream analyses. This is often controlled by constructing batches using a randomization procedure that balances key outcome variables (for example, case–control status) across batches. Ideally, randomization is performed when the full set of samples to be analyzed has been collected. Here, sample collection and sequencing occurred in parallel to rapidly generate data to study the host response to SARS-CoV-2 infection. To account for batch effects without masking signal of interest, eight samples were chosen as ‘batch controls’ to be included in every sequencing batch of 192 samples. Batch controls were manually selected to be a representative subset of the full cohort of samples with respect to key technical (for example, RNA quality) and biological (for example, COVID-19 status) variables.

### Randomization of RNA-seq batches

After selecting batch controls, samples were randomized into batches for RNA-seq (batch size of 192) and extraction (batch size of eight created within each sequencing batch of 192). One million permutations of batch assignments were performed. The permutation that was selected minimized the mean canonical correlation between batch assignment and the following set of clinical and demographic variables obtained from EHRs and sample variables: age, sex, race, ethnicity, COVID-19 status, deceased flag, ICU status, ventilation status, intubation status, timepoint, batch control status and blood volume collected. Randomization was done in multiple phases during sample collection, each phase performed on the set of samples that had been collected since the previous phase, thereby maximizing the degree of randomization that could be achieved while sequencing in parallel with sample collection. Batch control samples were included in the randomization but were forced to be present in every sequencing batch.

### RNA extraction, library preparation and sequencing

RNA extraction, library preparation and sequencing were performed as described previously^28^. In brief, frozen blood samples were thawed, and total RNA was extracted using a modification of the MagMax protocol for Stabilized Blood Tubes RNA Isolation Kit (Thermo Fisher Scientific, 4451893). Samples yielding sufficient RNA (>50 ng) were barcoded and prepared for pooled whole transcriptome sequencing using the TruSeq Stranded Total RNA Library Prep Gold (Illumina, 20020599), which is designed to remove ribosomal, globin and mitochondrial RNA. Libraries were amplified with 15 cycles of PCR, pooled and sequenced on a NovaSeq 6000 (Illumina) using Sprime flow cells with 100-bp paired-end reads, targeting a mean of 50 million read pairs per sample. For a minority of samples, the first extraction failed (*n* = 24), and RNA was re-extracted from the supernatant saved from the first centrifugation pellet. The extraction protocol was repeated starting with the second wash step after re-pelleting the RNA.

### Alignment and quantification of RNA-seq reads

After RNA-seq data collection, base calls were converted into raw reads and filtered after quality assessment. Quality-filtered raw data were converted into FASTQ files using bcl2fastq (Illumina). RNA-seq reads were aligned to the GRCh38 primary assembly with GENCODE gene annotation version 30 by STAR (version 2.7.3a)^60^ using per-sample two-pass mapping (–twopassMode Basic) and chimeric alignment options (–chimOutType Junctions SeparateSAMold -chimSegmentMin 15 -chimJunctionOverhangMin 15). RNA-seq quality control metrics were calculated by fastqc (version 0.11.8) and Picard Tools (version 2.22.3). Quantification was done at the gene level with antisense specificity using featureCounts (Subread R package version 1.6.3 and strandness option -s 2)^61^ with gene-level grouping / primary alignments only / count all overlapped features (-t exon -g gene_id -primary -O). MultiQC was used to compile and summarize per-sample statistics into an interactive HTML report^62^.

### Sample mislabeling correction

We assembled several sources of information to enable identification of mislabeled samples and inference of correct labels. For each RNA-seq sample, we defined expressed sex based on the relative abundance of the sex-specific genes *UTY* (male) and Xist (female). NGSCheckMate was used to determine which RNA-seq and WGS samples were empirically derived from the same individual based on correlation between variant allele fractions at a set of pre-specified loci (genetic match)^25^. These data were used to identify discrepancies between label matches and genetic matches and infer correct individual labels. In cases where mislabeling was present but not unambiguously correctable from the RNA-seq and WGS, the ambiguity was resolved by identifying samples that showed aberrant patterns in ELISA and Olink data, such as a single ELISA sample with substantially lower titers than both the previous and next samples from the same individual, or an Olink sample that failed to cluster with other samples from the same individual when visualized in Clustergrammer. In most cases, correct labels could be unambiguously inferred, even in complex cases involving multiple overlapping mislabeling events. Any mislabeled samples for which correct labels could not be inferred were discarded from all analyses.

### RNA-seq count data processing

DV200, the percentage of fragments longer than 200 nucleotides, has been shown to be more reliable than RNA integrity number to assess quality in RNA-seq data^26^. We, therefore, excluded samples with DV200 below 80% as well as samples with fewer than 10 million mapped reads counted by featureCounts. Despite globin depletion during library preparation, some samples showed substantial read counts for globin genes. To remove the unwanted signal due to globin gene expression in whole blood, counts for all annotated globin genes (gene symbols *CYGB*, *HBA1*, *HBA2*, *HBB*, *HBD*, *HBE1*, *HBG1*, *HBG2*, *HBM*, *HBQ1*, *HBZ* and *MB*) were discarded, and the remaining count matrix was transformed to counts per million (CPM). Genes with CPM ≥ 1 in ≥36 samples (half the number of individuals with no positive PCR or antibody test for SARS-CoV-2 during the study period) were included in our analyses (21,194). Gene expression was normalized for composition bias using the trimmed mean of M-values method, implemented by calcNormFactors in the edgeR package^63^ and transformed to normalized log_2_ CPM with observation weights computed by voomWithDreamWeights from the variancePartition package^29^.

RNA-seq data often contain technical and biological sources of variation irrelevant to the question at hand. Exploration of variance in the gene expression data was performed with principal component analyses (PCA, prcomp R function) and variance partitioning analyses^23^. Starting from normalized counts, we identified the variable that was the next strongest driver of unwanted variance, adjusted for this variable using linear modeling along with all previously selected ones and repeated this procedure iteratively until no more confounding variables were observed to be strong drivers of variance in the data. This resulted in a set of non-redundant technical and biological covariates explaining a substantial fraction of unwanted variation in the gene expression. To further identify covariates that might have an important impact on the gene expression data in a way that could not be easily captured by these analyses, we leveraged WGCNA co-expression network analyses^24^. We started by fitting a linear mixed model to the log_2_ CPM values including all previously selected variables using dream^29^ and extracted the residuals from this model (residualization). We then built a co-expression network from the residualized expression values and selected a new variable that was significantly correlated to many module eigengenes after multiple testing correction (Bonferroni-adjusted *P* < 0.05). The data were then residualized again, including the newly selected variable in the model, and the process was repeated until no more confounding variables were observed driving substantial variation in any modules.

Using this approach, we identified a set of technical and biological confounding variables that we used for all analyses performed. Specifically, whenever fitting a linear mixed model, we accounted for the following numeric covariates as fixed effects: number of days since the first blood sample, RNA DV200, age, PCT_R2_TRANSCRIPT_STRAND_READS and PCT_INTRONIC_BASES, WIDTH_OF_95_PERCENT, as well as accounting for the following categorical covariates as random effects: individual ID and expressed sex. The number of days since the first blood sample was modeled as a smooth non-linear function defined using natural cubic splines (ns R function^64^) with internal knots at 1, 3, 7 and 12, reflecting the timeline of blood draws for individuals in our cohort. All other fixed effect technical variables were scaled to have a mean of 0 and a variance of 1 using the scale R function. The last three fixed effects listed are sequencing quality metrics computed by Picard Tools: PCT_R2_TRANSCRIPT_STRAND_READS is ‘the fraction of reads that support the model where R2 is on the strand of transcription, and R1 is on the opposite strand’; PCT_INTRONIC_BASES is the ‘fraction of PF_ALIGNED_BASES that correspond to gene introns’; and WIDTH_OF_95_PERCENT is the difference between the 2.5th percentile and the 97.5th percentile of the insert size distribution.

PCA was performed on the residualized expression matrix after adjusting for all covariates selected above using a linear mixed model for all samples, and outliers were removed by drawing an ellipse in the first two principal components (PCs) centered at the origin encompassing 3 standard deviations of PCs 1 and 2 and then discarding all samples outside this ellipse. Batch effects were residualized from the data by fitting a linear mixed model to the normalized log_2_ CPM and weights for each gene with random effects for library prep plate (the batch effect to be residualized out) and blood sample ID (the biological signal to be retained) using dream^29^, and then subtracting the best linear unbiased predictors for library prep plate while retaining the differences between blood samples and the residuals. Then, the technical replicates for each batch control sample were summarized to a single residualized expression value equal to the weighted mean of the technical replicates with a weight equal to the sum of the individual weights of the technical replicates. This yielded a batch-residualized expression matrix with 21,194 rows (genes) and 1,392 columns (blood samples) and a corresponding matrix of observation weights that was used as the input for all differential expression testing.

### Cell type deconvolution and validation

Cell type fractions were estimated for each sample using CIBERSORTx^65^, providing transcripts per million (TPM) as input, following procedures recommended by the documentation, and pooling reads from all technical replicates when computing TPM for batch control samples. CIBERSORTx requires a reference dataset to determine the set of possible cell types as well as the set of CTS genes that will be used for deconvolution. To ensure the most accurate estimation, we tested four independent references generated by different labs with different technologies. The LM22 reference consists of bulk RNA-seq data from PBMCs sorted by fluorescence-activated cell sorting, whereas the NSCLC PBMC, SCP424 and Wilk references are derived from single-cell RNA-seq of PBMCs in various disease contexts^27,66,67^. The SCP424 dataset was pre-processed as previously described^28^. Marker genes for the Wilk reference were defined as those upregulated in each of 20 different cell types (relative to the other 19) with adjusted *P* value below 0.05 (ref. ^66^).

For validation, the cell type fractions estimated with each reference were grouped into neutrophils, monocytes and lymphocytes and summed within groups, and then Pearson correlation was computed between each group’s fractions and the corresponding complete blood count fraction recorded on the day of sample collection. The cell types for LM22, NSCLC PBMC and SCP424 were grouped as described previously^28^, whereas the groupings for the Wilk reference were as follows: for monocytes, ‘CD14 Monocyte’ and ‘CD16 Monocyte’; for lymphocytes, ‘CD8m T’, ‘CD4m T’, ‘B’, ‘IFN-stim CD4 T’, ‘Proliferative Lymphocytes’, ‘γδ’, ‘IgM PB’, ‘IgG PB’ and ‘IgA PB’; and for neutrophils, just ‘Neutrophil’. The LM22 reference cell type fractions had consistently the highest correlation and were used for all further analyses involving cell type fractions.

### Cell type selection

To control for variations in cell type fractions between samples, we identified a minimal set of cell type fractions explaining the variation in severity, to include as covariates when fitting models for differential expression. We used glmmLasso to fit an L1-penalized ordinal regression generalized linear mixed model to all estimated cell type fractions while controlling for the identified confounders, with severity as the response using the adjacent categories family (glmmLasso function with options family = acat(), final.re = TRUE and switch.NR = TRUE)^68^. We optimized the tuning parameter lambda by a grid search from 0 to 500 counting by 5 and found that Bayesian information criterion was minimized at lambda = 95. Finally, we determined a set of non-redundant cell types explaining severity by selecting all cell types with non-zero parameters after penalization.

To select the cell types to be tested in the cell fraction interaction model described below, we used variancePartition to determine the contribution of COVID-19 severity to the variation observed in each cell type by running fitVarPartModel and extractVarPart on the cell type fractions with a model including all identified confounders as well as severity as a random effect^23^. Cell types in which severity explained at least 1% of variance and which had non-zero fractions in at least 20% of samples were selected for DE testing with the interaction model.

### DE analyses

For each PASC checklist item, we fit a linear mixed model to the batch-corrected expression of each gene, controlling for all previously identified confounders, COVID-19 severity at the time of sampling and any ICU encounter during their hospital stay as random effects and selected cell type fractions as fixed effects. We tested each gene for differences in expression between the ‘true’ and ‘false’ groups using dream^29^. In addition, for each combination of checklist item and cell type, we fit the same model with an additional fixed effect interaction term between the checklist item and the cell type fraction. This interaction model estimates, for each group of the checklist item, a coefficient for the slope of gene expression with respect to the specified cell type fraction in that group. We tested for differences in these slope coefficients for the ‘true’ and ‘false’ groups, therefore looking for genes whose expressions are varying with the cell type fraction in different ways between groups. The scale of log_2_ fold change (logFC) values reported from this interaction model is dependent on factors such as the range of cell type fractions observed for the specified cell type and, thus, cannot be simply interpreted as in a typical difference-of-means model. However, the signs, relative differences, measures of effect size and statistical significance have the same meaning as they would for a typical mean difference coefficient. We, therefore, report standardized logFC values (normalized using the R function scale with center = 0) that are more directly interpretable. Lastly, we tested for differences in gene expression that are independent of the antibody response to the SARS-CoV-2 spike protein by fitting all models a second time with three additional coefficients controlling for the log_2_ titers of anti-spike protein IgG, IgA and IgM. For this, we included all 1,301 samples from 543 individuals who had both RNA-seq and serology measures (329 samples from 158 individuals with PASC checklists). For each DE test, we controlled for multiple testing among the 21,194 genes tested using the Benjamini–Hochberg method. We focused our downstream analyses on cell type/symptom combinations for which at least 100 genes were DE at false discovery rate (FDR) ≤ 0.05.

Alternate DE models to evaluate consistency of results on biological covariate selection were generated as follows. For each combination of cell type and symptom, we fit several DE models while including or omitting specific biological covariates. First, for each of expressed sex, age, severity and ICU encounter, an alternate model was fit in which the specified biological covariate was omitted (that is, not controlled for). Other than the omission of the specified covariate, all other variables remained the same. We evaluated the consistency of DEGs between each alternate model and the corresponding original model by performing one-sided Fisher’s exact test for enrichment of same-direction DEGs, adjusted for multiple testing using the Benjamini–Hochberg method. No substantial differences in DE results were observed in these alternate models, likely because controlling for individual ID adequately accounts for inter-individual variation explained by these covariates. In particular, the total number of DEGs in each alternate model remained similar to the original (Extended Data Fig. 4b), and the DEGs detected in alternate models were always significantly overlapped with the DEGs from the original model in a consistent direction in every case where the original model had at least 100 DEGs (all Fisher’s exact test ORs ≥ 5,775, adjusted *P* ≤ 1.98 × 10^−130^)^.^

Next, for each of IgA, IgG and IgM, an alternate model was fit controlling for the anti-spike antibody titer of that single class only. These models were evaluated for consistency with the models controlling for all three titers as described above. In almost every case, controlling for any one class of antibody gave almost the same result as controlling for all three classes, with similar numbers of DEGs and significant overlap of DEGs in a consistent direction (Extended Data Fig. 4a, all Fisher’s exact test ORs ≥ 2,245, adjusted *P* ≤ 9.32× 10^−222^), indicating that the anti-spike antibody dependence of CTS DEGs are not specific to any one class.

### Validation of cell type interaction model in independent pseudo-bulk dataset

A single-cell RNA-seq dataset from an independent cohort of patients with COVID-19 who were assessed for PASC^16^ was used to validate the cell type interaction model used for CTS DE testing. Cell type fractions were computed for each sample by dividing the cell counts for each cell type by the total number of cells for the sample. ‘Whole blood’ pseudo-bulk (PB) read counts were computed for each sample by summing the read counts for all cells in a sample. CTS PB read counts were computed for each sample by summing the read counts for each of the five major cell types described in the original work presenting the data^16^ (‘B_cells’, ‘CD4_T_cells’, ‘CD8_T_cells’, ‘Monocytes’ and ‘NK_cells’). In each PB count matrix, the average log_2_ CPM was computed for each gene across all samples using the aveLogCPM function in the edgeR package^63^, and the median was computed across all genes and used as the abundance threshold for filtering that count matrix. Genes with log_2_ CPM values above the chosen threshold in at least 10% of all samples were included in the DE tests described below. Filtered PB count matrices were each normalized for composition bias using the trimmed mean of M-values method^63^ and transformed to normalized log_2_ CPM as described in the ‘RNA-seq count data processing’ section of the Methods (ref. ^29^), with a design including all biological and technical covariates described below.

All DE tests performed on PB data controlled for the following biological and technical covariates, chosen to match those used in the bulk RNA-seq DE analyses as closely as possible: COVID-19 severity, ICU admission during acute COVID-19, encounter location (home, clinic or hospital), individual ID, age, sex and timepoint (T1, T2 or T3). Age was modeled as a fixed effect, whereas all other listed variables were modeled with random effects. COVID-19 severity was defined based on the provided World Health Organization ordinal scale^69^ values as follows: mild, 2 or less; moderate, 3 or 4; severe, 5 or 6; and severe with EOD, 7. Each PASC symptom was tested for CTS DE in the whole blood PB data using the cell type interaction model described in the ‘Differential expression analyses’ section of the Methods. Cell type composition was accounted for by selecting three of the four remaining cell types and adding their corresponding cell fractions as covariates. Other than the cell type being tested, the three cell types with the highest median fractions across all samples were included in each model. Each PASC symptom was also tested for CTS DE by analyzing the CTS PB data without controlling for whole blood cell type composition. In addition, because T3 is a post-acute timepoint in this dataset, a ‘phase’ variable was defined as ‘acute’ for T1 and T2 and ‘post-acute’ for T3. In all DE analyses of PB data, this ‘phase’ variable was added as an interaction term, and DE tests were performed only on the coefficients specific to the acute phase gene expression.

To evaluate the consistency of the signal identified by these two methods of testing for CTS DE, Spearman correlations were computed between the logFC values for every whole blood interaction model and the logFC values for every CTS PB model. Correlations were divided into matching (same cell type and same symptom in both models) and non-matching (different cell type or symptom between models) groups. One-sided Student’s *t*-tests were performed for the following alternative hypotheses: matching correlations are greater than non-matching correlations; matching correlations are greater than zero; and non-matching correlations are greater than zero. The matching correlations were significantly greater than zero (*t* = 11.8, *P* = 3.11 × 10^−23^) and significantly greater than non-matching correlations (*t* = 11.7, *P* = 3.99 × 10^−23^), whereas the latter were not significantly greater than zero (*t* = 0.295, *P* = 0.384), validating our interaction model as one that can accurately detect CTS DE.

Although this dataset was useful for validating the ability of our modeling strategy to quantify CTS expression, direct comparison of DEGs between datasets was not possible owing to substantial population and methodological differences between the two datasets, such as the inclusion of non-hospitalized individuals, the very different time of assessment for PASC (2–3 months after onset of acute symptoms in the independent dataset versus ~1 year after discharge) and the lack of clear matches between symptom definitions.

### Titer-stratified DE models

To validate the computational inference of antibody dependence of DE signatures, we conducted DE while stratifying by titers rather than controlling for them. Samples were stratified by the maximum titer of anti-spike IgG, IgA and IgM, defining high-titer and low-titer strata as the top and bottom 30% of samples, respectively (the middle 40% was not used for this analysis). The RNA-seq DE model for each combination of symptom and cell type was fit separately to the high-titer and low-titer strata without controlling for antibody titers. Although these strata contain too few samples to reliably detect individual DEGs, and the Spearman correlations between the logFCs of the two strata are too weak to be informative, we hypothesized that the Spearman correlations of each stratum’s logFCs against the logFCs from the full data would be informative, because the full dataset is well-powered. Specifically, assuming the low-titer and high-titer strata have similar power to detect DE, when DE is antibody-independent, correlations to the full data should be equal for both strata, because these logFCs are, by assumption, not driven by antibody titers. Conversely, when DE is antibody-dependent, one stratum should correlate more highly than the other, because the logFCs are influenced by the antibody titers and, therefore, different between the low-titer and high-titer strata. Spearman correlations were computed for the logFCs from these stratified models against the logFCs from the corresponding model fit to the full dataset without adjustment for anti-spike antibody titers, and the absolute difference was computed between these correlations of the high-titer model and the low-titer model (referred to as ‘absolute correlation difference’). For plasma cells, we see a significantly higher mean absolute correlation difference (one-sided Student’s *t*-test, *t*(3) = 3.45, *P* = 0.021) when comparing nausea/diarrhea/vomiting, sleep_problems and smell/taste problems (antibody-dependent, mean absolute correlation difference = 0.33) against lung problems and skin rash (antibody-independent, mean absolute correlation difference = 0.11), recapitulating our inference of titer-dependence from the original model.

### Shared DEG analysis

We defined the same-direction shared DEGs between two DE tests to be the set of genes that are differentially expressed in both tests and have the same sign on their logFCs (that is, both negative or both positive). Similarly, we defined the opposite-direction shared DEGs as the set of genes differentially expressed in both tests but with opposing signs (that is, negative in one test and positive in the other). We tested for enrichment in shared DEGs by performing a one-sided Fisher’s exact test for enrichment of either the same-direction or opposite-direction shared DEGs among all the DEGs for each of the two DE tests. We controlled for multiple testing using Holm’s method for family-wise error rate (FWER) control among all comparisons performed within a given symptom or cell type between DE signatures with at least 100 DEGs either before or after controlling for anti-spike antibody titers.

### GO term enrichment analyses for DE signatures

For each DE test, downregulated and upregulated DEGs were separately tested for GO term enrichment for all GO terms annotated to at least ten expressed genes, using the Bioconductor packages goseq, topGO and org.Hs.eg.db. A one-sided Fisher’s exact test was performed for the enrichment of the upregulated or downregulated DEGs for each GO term, with all 21,194 expressed genes as the background. For each enrichment analysis, we controlled for multiple testing among all GO terms tested using the Benjamini–Hochberg method.

### Enrichment tests of CTS DEGs for cell type marker genes

To verify that the interaction models between PASC checklist items and cell type fractions captured CTS DEGs, we tested for enrichment of CTS marker genes. For each of several broad cell type categories (Supplementary Table 6), we assembled a set of marker genes from the literature as the union of all marker genes for each category^66,70–72^. We defined the list of DEGs for each category as the union of the DEGs from all PASC symptoms for all LM22 cell types in that category and tested whether this union of DEGs was enriched for the marker genes using a one-sided Fisher’s exact test. *P* values were adjusted for multiple testing using the Benjamini–Hochberg method.

### Linear mixed models for serology, acute phase CDE and cell fractions

For each estimated cell type fraction, we tested for differences between the ‘true’ and ‘false’ groups of each checklist item using dream^29^ in the same manner as for the gene expression data, except that coefficients for the cell type fractions themselves were omitted from the model. Likewise, we tested for differences in each CDE measured during hospitalization, omitting the coefficients for the cell type fractions and all RNA-related coefficients (RNA DV200, PCT_R2_TRANSCRIPT_STRAND_READS, PCT_INTRONIC_BASES and WIDTH_OF_95_PERCENT). Lastly, we tested for differences in log_2_ titers of anti-spike IgG, IgA and IgM using the same model as for the CDE.

### Validation of anti-spike antibody titer associations with PASC

Acute anti-spike antibody titers for IgA and IgG were measured in an independent, previously published dataset of patients with COVID-19 with post-acute follow-up^17^. For each individual in this dataset, the anti-spike titers were grouped by whether the individual later developed any PASC symptom, and a two-sided Mann–Whitney test was performed between the two groups for each antibody.

### Validation of anti-spike antibody independent associations of total antibody titer to PASC

In an independent dataset of patients with COVID-19 with post-acute follow-up^17^, a linear model was fit for the prediction of the product of total IgM and total IgG3 with coefficients for PASC, anti-spike IgA, anti-spike IgG, severe acute COVID-19, ICU admission during acute COVID-19, sex and age, using the following formula:

$$\begin{array}{l}IgM \times IgG3\sim PASC + anti\,spike\,IgA + anti\,spike\,IgG\\ + Severe\,Acute\,COVID19 + ICU\,admission + sex + age\end{array}$$

Coefficient point estimates, confidence intervals and *P* values were computed in R using the lm function^64^. Additionally, a logistic regression model for prediction of PASC was fit with coefficients for total IgM, total IgG3, IgM*IgG3, anti-spike IgA, anti-spike IgG, number of acute symptoms, history of asthma bronchiale and age, using the following formula:

$$\begin{array}{l}PASC\sim IgM \times IgG3 + anti\,spike\,IgA + anti\,spike\,IgG\\ + number\,of\,acute\,symptoms + history\,of\,asthma\,bronchial + age\end{array}$$

Coefficient point estimates, confidence intervals and *P* values were computed in R using the glm function as described previously^17,64^.

### Other data processing, analyses and visualization

CDEs were queried from Epic Clarity, Epic Caboodle and several in-house databases. Manual chart review was conducted using Epic Hyperspace. Most data analyses were performed using the R statistical language major version 4 (ref. ^73^) and the Bioconductor suite of packages^74^. Large data tables were read, written and processed using many tidyverse packages^75^ as well as the R package data.table. With the exceptions of Extended Data Figs. 3a and 5, all plots were created using ggplot2 (ref. ^76^). The following statistical tests and methods were implemented by R functions unless otherwise noted: Student’s *t*-tests: t.test; Mann–Whitney tests (also known as Wilcoxon rank-sum tests): wilcox.test; Fisher’s exact tests: fisher.test; tests of correlation: cor.test; fixed effects linear models: lm^64^; and multiple testing correction: p.adjust R function (specific adjustment methods noted above). Analyses were run in parallel using the R packages future, BiocParallel, foreach, doMC, batchtools^77^ and parallelDist. Intermediate results were cached for faster re-analysis using the R packages memoise and cachem. Study data were collected and managed using REDCap electronic data capture tools hosted by Scientific Computing at the Icahn School of Medicine at Mount Sinai^78^. Rows and columns in correlation plots and shared DEG plots were ordered using the R package seriation^79^. GO term enrichment results were clustered with GO-Figure!^30^ and visualized using the R package treemap. GO-Figure! and the first step of NGSCheckMate (variant allele fraction calculation) were run using Python 3.7.3. The 2nd step of NGSCheckMate (computation of inter-sample correlations and other statistics) was rewritten in R to accommodate larger sample sizes. Canonical correlations among all technical, clinical and demographic variables were calculated using the canCorPairs function and visualized using the plotCorrMatrix function from the Bioconductor package variancePartition^23^. CIBERSORTx was run using Singularity.

### Reporting summary

Further information on research design is available in the Nature Portfolio Reporting Summary linked to this article.

## Online content

Any methods, additional references, Nature Portfolio reporting summaries, source data, extended data, supplementary information, acknowledgements, peer review information; details of author contributions and competing interests; and statements of data and code availability are available at 10.1038/s41591-022-02107-4.

## Supplementary information

Supplementary Information Legends for Extended Data Figs. 1–8 and data descriptions for Supplementary Data Tables 1–6.

Reporting Summary

Supplementary Data 1 S1A Full Cohort Description: Population description for full cohort of 232 subjects. S1B RNA-seq Cohort Description: Population description for core cohort of 165 subjects with RNA-seq data. S1C Symptoms vs. Clinical Data: Table of tests for dependence of PASC symptoms on medications, labs and comorbidities. S1D Symptoms vs. Anti-Spike Ab: Table of tests for dependence of PASC symptoms on anti-spike antibody titers. S1E Symptoms vs. Cell Type Fraction: Table of tests for dependence of PASC symptoms on estimated cell type fractions.

Supplementary Data 2 S2A PASC DEGs, no titer adj.: Table of significant DEGs (adj.P.Val ≤ 0.05) for all PASC symptoms and cell types, with no adjustment for anti-spike antibody titers (ModelVariant = ‘Original’). S2B PASC DEGs, titer-adjusted: Table of significant DEGs (adj.P.Val ≤ 0.05) for all PASC symptoms and cell types, with adjustment for anti-spike antibody titers of IgA, IgG and IgM (ModelVariant = ‘Serology_AGM’).

Supplementary Data 3 S3A B cells mem. NoTA: Table of significantly enriched (adj.P.Val ≤ 0.05) Gene Ontology terms for B cells memory for all PASC symptoms without adjustment for anti-spike antibody titers. S3B Macrophages M0 NoTA: Table of significantly enriched (adj.P.Val ≤ 0.05) Gene Ontology terms for Macrophages M0 for all PASC symptoms without adjustment for anti-spike antibody titers. S3C Macrophages M1 NoTA: Table of significantly enriched (adj.P.Val ≤ 0.05) Gene Ontology terms for Macrophages M1 for all PASC symptoms without adjustment for anti-spike antibody titers. S3D Mast cells rest NoTA: Table of significantly enriched (adj.P.Val ≤ 0.05) Gene Ontology terms for Mast cells resting for all PASC symptoms without adjustment for anti-spike antibody titers. S3E Monocytes NoTA: Table of significantly enriched (adj.P.Val ≤ 0.05) Gene Ontology terms for Monocytes for all PASC symptoms without adjustment for anti-spike antibody titers. S3F Neutrophils NoTA: Table of significantly enriched (adj.P.Val ≤ 0.05) Gene Ontology terms for Neutrophils for all PASC symptoms without adjustment for anti-spike antibody titers. S3G NK cells rest NoTA: Table of significantly enriched (adj.P.Val ≤ 0.05) Gene Ontology terms for NK cells resting for all PASC symptoms without adjustment for anti-spike antibody titers. S3H Plasma cells NoTA: Table of significantly enriched (adj.P.Val ≤ 0.05) Gene Ontology terms for Plasma cells for all PASC symptoms without adjustment for anti-spike antibody titers. S3I T cells CD4 mem. act. NoTA: Table of significantly enriched (adj.P.Val ≤ 0.05) Gene Ontology terms for T cells CD4 memory activated for all PASC symptoms without adjustment for anti-spike antibody titers. S3J T cells CD4 mem. rest NoTA: Table of significantly enriched (adj.P.Val ≤ 0.05) Gene Ontology terms for T cells CD4 memory resting for all PASC symptoms without adjustment for anti-spike antibody titers. S3K T cells CD8 NoTA: Table of significantly enriched (adj.P.Val ≤ 0.05) Gene Ontology terms for T cells CD8 for all PASC symptoms without adjustment for anti-spike antibody titers. S3L T cells gamma delta NoTA: Table of significantly enriched (adj.P.Val ≤ 0.05) Gene Ontology terms for T cells gamma delta for all PASC symptoms without adjustment for anti-spike antibody titers. S3M B cells mem. TA: Table of significantly enriched (adj.P.Val ≤ 0.05) Gene Ontology terms for B cells memory for all PASC symptoms with adjustment for anti-spike antibody titers. S3N Macrophages M1 TA: Table of significantly enriched (adj.P.Val ≤ 0.05) Gene Ontology terms for Macrophages M1 for all PASC symptoms with adjustment for anti-spike antibody titers. S3O Monocytes TA: Table of significantly enriched (adj.P.Val ≤ 0.05) Gene Ontology terms for Monocytes for all PASC symptoms with adjustment for anti-spike antibody titers. S3P Neutrophils TA: Table of significantly enriched (adj.P.Val ≤ 0.05) Gene Ontology terms for Neutrophils for all PASC symptoms with adjustment for anti-spike antibody titers. S3Q NK cells rest TA: Table of significantly enriched (adj.P.Val ≤ 0.05) Gene Ontology terms for NK cells resting for all PASC symptoms with adjustment for anti-spike antibody titers. S3R Plasma cells TA: Table of significantly enriched (adj.P.Val ≤ 0.05) Gene Ontology terms for Plasma cells for all PASC symptoms with adjustment for anti-spike antibody titers. S3S T cells CD4 mem. act. TA: Table of significantly enriched (adj.P.Val ≤ 0.05) Gene Ontology terms for T cells CD4 memory activated for all PASC symptoms with adjustment for anti-spike antibody titers. S3T T cells CD4 mem. rest TA: Table of significantly enriched (adj.P.Val ≤ 0.05) Gene Ontology terms for T cells CD4 memory resting for all PASC symptoms with adjustment for anti-spike antibody titers. S3U T cells CD8 TA: Table of significantly enriched (adj.P.Val ≤ 0.05) Gene Ontology terms for T cells CD8 for all PASC symptoms with adjustment for anti-spike antibody titers. S3V T cells gamma delta TA: Table of significantly enriched (adj.P.Val ≤ 0.05) Gene Ontology terms for T cells gamma delta for all PASC symptoms with adjustment for anti-spike antibody titers.

Supplementary Data 4 S4A PASC DEGs, term elim.: Table of significant DEGs (adj.P.Val ≤ 0.05) for all PASC symptoms and cell types, with specific terms dropped from the model (ModelVariant ‘No*’). S4B PASC DEGs, 1-titer adj.: Table of significant DEGs (adj.P.Val ≤ 0.05) for all PASC symptoms and cell types, with adjustment for single classes of anti-spike antibody titers (ModelVariant = ‘Serology_Ig*’).

Supplementary Data 5 S5A No Serology Adjustment: Cell type marker enrichment results for CTS DEGs with no serology adjustment. S5B Serology Adjustment: Cell type marker enrichment results for CTS DEGs with serology adjustment.

Supplementary Data 6 S6A DEG Cell Type mappings: Table of cell type mappings from LM22 reference to broad categories used to assemble lists of CTS DEGs. S6B Marker Gene Cell Type Map: Table of cell type mappings used to annotate cell type marker genes to broad categories from the scientific literature.

## Data Availability

All data, methods and materials for the Mount Sinai COVID-19 Biobank are available in the main text, in the Methods, in the Supplementary Information or via Synapse project ID syn35874390. Researchers may access the data on Synapse after registering for a free account. There are no other restrictions on access or use of these data. The Synapse project includes directions for accessing the RNA-seq gene expression data, which are available on the National Center for Biotechnology Information Gene Expression Omnibus under accession number GSE215865, along with instructions for linking these data with the corresponding data (clinical, technical and other) on Synapse. Validation data were obtained directly from the authors of previously published work^16,17^. Source data are provided with this paper.

## Source data

Source Data Fig. 2 Statistical Source Data for Fig. 2a
Source Data Fig. 4 Statistical Source Data for Fig. 4
Source Data Extended Data Fig. 1 Statistical Source Data for Extended Data Fig. 1
Source Data Extended Data Fig. 6 Statistical Source Data for Extended Data Fig. 6

## Code availability

A modified version of NGSCheckMate suitable for large datasets is available at https://github.com/DarwinAwardWinner/NGSCheckMate. Additional supporting code is available at https://github.com/DarwinAwardWinner/rctutils. No other custom code was used.
