# Supplementary material for: Molecular states during acute COVID-19 reveal distinct etiologies of long-term sequelae
Source: Nat Med. 2022 Dec 8;29(1):236–46. doi: 10.1038/s41591-022-02107-4 (PMC9873574; doi:10.1038/s41591-022-02107-4)
Supplement: Supplementary file 1 — Legends for Extended Data Figs. 1–8 and data descriptions for Supplementary Data Tables 1–6. [file 41591_2022_2107_MOESM1_ESM.pdf]

nature medicine

Article https://doi.org/10.1038/s41591-022-02107-4

Molecular states during acute COVID-19 reveal distinct etiologies of long-term sequelae

In the format provided by the authors and unedited

Table of Contents

Extended Data Figures *2*

Extended Data Figure 1: Correlation of occurrences of PASC checklist items, comorbidities, demographics, and acute disease metrics **2**

Extended Data Figure 2: Relationship between acute anti-spike antibody titers and PASC with independent validation **3**

Extended Data Figure 3: Cell-type fraction estimations and interaction model **4**

Extended Data Figure 4: Cell-type-specific differential expression for PASC checklist items (full) **5**

Extended Data Figure 5: GO enrichments for DEGs for other PASC checklist items. **6**

Extended Data Figure 6: Shared PASC checklist DEGs between cell-types **7**

Extended Data Figure 7: Delta-MA plot of anti-spike antibody titer effect on differential expression log (fold change) **8**

Extended Data Figure 8: PASC prediction by total Ig in independent data set is independent of anti-spike Ig **9**

Supplementary Data Description *10*

Supplementary Table 1: Cohort description and clinical associations to PASC checklist items **10**

Supplementary Table 2: DEGs before and after controlling for anti-spike antibody titers **10**

Supplementary Table 3: GO term enrichments for PASC symptoms DE signatures **10**

Supplementary Table 4: DEGs for single-titer and term-elimination alternative models .. **12**

Supplementary Table 5: Marker gene enrichments in cell-type-specific DEGs **12**

Supplementary Table 6: Cell-type mapping for marker gene enrichments **12**

Extended Data Figures

Extended Data Figure 1: Correlation of occurrences of PASC checklist items, comorbidities, demographics, and acute disease metrics

The axes are representative of the symptoms, comorbidities, demographics, and acute disease metrics, and the color represents the Pearson correlation of their coincidence. Comorbidities present before COVID-19 hospitalization are defined with the prefix “prior” in the axis label. Correlations with family wise error rate (FWER, Holm 1979) adjusted p-values < 0.05 (2-sided Fisher’s exact test) are indicated with a star. Rows and columns are ordered by hierarchical clustering and optimal leaf ordering.

Extended Data Figure 2: Relationship between acute anti-spike antibody titers and PASC with independent validation

a) Association of acute anti-spike antibody titers to PASC symptoms in Mount Sinai cohort. The y axis is the symptom assessed, and the x axis is the p-value ($-\log_{10}$) for the association of the anti-spike antibody titers to the symptoms (linear mixed model, 2-sided *t*-test). The shape indicates the class of anti-spike antibody tested, and the color indicates whether the association is significant (BH FDR ≤ 0.05)

b) Independent dataset validation of the non-association of acute anti-spike antibody titers to PASC. The x axis is the anti-spike antibody class and the y axis the titer measured for antibodies against the S1 domain of the spike protein during acute COVID-19. Each point represents an individual (n = 134 individuals, 85 with PASC). The color indicates the presence and absence of PASC and is defined in the legend. Two sided Mann-Whitney test unadjusted p-values are shown between groups indicated by the brackets. Distributions are shown using box-and-whiskers plots (thick bar, median; box, 25th to 75th percentile, whiskers reach to the largest/smallest observations within 1.5 box-heights of the box.

<u>Extended Data Figure 3: Cell-type fraction estimations and interaction model</u>

a) Validation heatmap of estimated cell-type fractions with clinical complete blood counts. The x axis shows the literature reference dataset used for the deconvolution procedure and the y axis is the cell-type fractions validated. The colors represent the Pearson correlation (rho) values between the estimated cell-type fractions and the corresponding complete blood count from the clinical data. The correlation values and associated 2-sided p-values adjusted for multiple testing (FWER, Holm 1979) and are noted in each box. Some reference data sets did not include neutrophils (indicated by gray boxes). b) Estimated cell-type fraction variance explained by biological and technical variables. The x axis is the percent of variance of the cell-type fractions explained by covariates (colors) and the y axis the cell type assessed. Cell types are ordered by the decreasing percent of their variance explained by COVID-19 severity. The black dashed line represents the cutoff for inclusion in the cell-type-specific analyses. c) Schematic of interaction model for mock genes A and B. The x axis is the cell-type fraction of a specific cell-type of interest and the y axis the gene expression in $\log_2$(counts per million). The color represents the presence (red) and absence (blue) of a symptom. The left and right facets show a gene not differentially expressed (same slope) and a differentially expressed gene (different slopes) respectively.

Extended Data Figure 4: Cell-type-specific differential expression for PASC checklist items (full)

The x axes are PASC checklist items (arranged in order of descending prevalence) and the y axes are the number of upregulated (above 0) and downregulated (below 0) DEGs at FDR≤0.05. Each row presents DE results for the indicated cell type. The dashed grey lines indicate the 100 DEG mark. The colors of the bars (defined in the legends) indicate (a) DE results for the specified anti-spike antibody titer adjustment (or no adjustment for titers, "None"), and (b) DE results when eliminating the specified term from the original model (shown as "None"). Note: "None" and "IgA+IgG+IgM" bars from Figure 3 are included here for ease of comparison.

Extended Data Figure 5: GO enrichments for DEGs for other PASC checklist items.

Box sizes are relative to the $-\log_{10}$(adjusted p-values) of the GO term enrichments for the corresponding DEGs and the term is noted in each box. Related terms are grouped by similarity and groupings are indicated by proximity and shared color. Consensus terms are indicated in bold for each group. a) Upregulated genes in memory resting $CD4^+$ T cells for cavities/teeth problems. b) Downregulated genes in $CD8^+$ T cells for quality of life. c) Upregulated genes in M1 macrophages for need supplemental O_2. d) Upregulated genes in memory B cells for anxiety/depression. e) Upregulated genes in memory activated $CD4^+$ T cells for memory/thought problems.

Extended Data Figure 6: Shared PASC checklist DEGs between cell-types.

The x and y axes are the cell types associated with more than 100 DEGs. The numbers in each box are the numbers of shared DEGs between the two checklist items defined in the axes, and the color represents whether they are same-direction (blue), opposite direction (red) or the total number of DEGs for that checklist item (grey). The shadings of red and blue are the ORs of the 1-sided Fisher's exact tests for the enrichment of shared DEGs in that box, and are shown only if the associated enrichment adjusted p-value < 0.05 (FWER, Holm 1979). The left and right facets represent the shared DEGs before and after adjustment for anti-spike antibody titers respectively. Symptoms in rows and columns are ordered by hierarchical clustering and optimal leaf ordering based on the shared same-direction DEGs. a) Quality of life shared DEGs. b) Cavities and teeth problems shared DEGs.

Extended Data Figure 7: Delta-MA plot of anti-spike antibody titer effect on differential expression log (fold change)

The x and y axes represent the average normalized gene expression and the differential expression log fold change (logFC) respectively. Each arrow shows a single DEG. The arrow colors indicate the anti-spike antibody titer dependent (red) and independent (blue) DEGs. The contours show the distribution of all logFC values before controlling for antibody titers. The effect of controlling for antibody titers on DEGs is shown by the arrows, with the arrow tail being the logFC before adjustment and the arrow head the logFC after adjustment. LogFC values in each panel are scaled such that the root mean square logFC before adjustment is equal to 1. a) Sleep problems in plasma cells. b) Nausea/diarrhea/vomiting in plasma cells. c) Smell/taste problems in plasma cells. d) Lung problems in plasma cells. e) Skin rash in plasma cells. f) Pneumonia in plasma cells. g) Anxiety/depression in memory B cells. h) Need Supplemental O_2 in M1 macrophages.

Extended Data Figure 8: PASC prediction by total Ig in independent data set is independent of anti-spike Ig

Plot of logistic regression model and p-values (2-sided likelihood ratio test, no adjustment for multiple testing) for prediction of PASC (n = 134 individuals, 85 with PASC). The y axis lists all non-intercept coefficients, and the x axis shows the coefficient values, with the black center point showing the fitted value and the error bars showing the 95% confidence interval (CI) about this value. CIs that include 0 are colored red, while those that indicate a significant difference from 0 ($p < 0.05$) are colored blue.

Supplementary Data Description

Supplementary Table 1: Cohort description and clinical associations to PASC checklist items

Description: Description of sheets and columns

S1A Full Cohort Description: Population description for full cohort of 232 individuals

S1B RNA-seq Cohort Description: Population description for core cohort of 165 individuals with RNA-seq data

S1C Symptoms vs. Clinical Data: Table of tests for dependence of PASC symptoms on medications, labs, and comorbidities

S1D Symptoms vs. Anti-Spike Ab: Table of tests for dependence of PASC symptoms on anti-Spike antibody titers

S1E Symptoms vs. Cell Type Fraction: Table of tests for dependence of PASC symptoms on estimated cell type fractions

Supplementary Table 2: DEGs before and after controlling for anti-spike antibody titers

Description: Description of sheets and columns

S2A PASC DEGs, no titer adj.: Table of significant DEGs (adj.P.Val ≤ 0.05) for all PASC symptoms and cell types, with no adjustment for anti-spike antibody titers (ModelVariant = "Original")

S2B PASC DEGs, titer-adjusted: Table of significant DEGs (adj.P.Val ≤ 0.05) for all PASC symptoms and cell types, with adjustment for anti-spike antibody titers of IgA, IgG, and IgM (ModelVariant = "Serology_AGM")

Supplementary Table 3: GO term enrichments for PASC symptoms DE signatures

Description: Description of sheets and columns

S3A B cells mem. NoTA: Table of significantly enriched (adj.P.Val ≤ 0.05) Gene Ontology terms for B cells memory for all PASC symptoms without adjustment for anti-spike antibody titers.

S3B Macrophages M0 NoTA: Table of significantly enriched (adj.P.Val ≤ 0.05) Gene Ontology terms for Macrophages M0 for all PASC symptoms without adjustment for anti-spike antibody titers.

S3C Macrophages M1 NoTA: Table of significantly enriched (adj.P.Val ≤ 0.05) Gene Ontology terms for Macrophages M1 for all PASC symptoms without adjustment for anti-spike antibody titers.

S3D Mast cells rest NoTA: Table of significantly enriched (adj.P.Val ≤ 0.05) Gene Ontology terms for Mast cells resting for all PASC symptoms without adjustment for anti-spike antibody titers.

S3E Monocytes NoTA: Table of significantly enriched (adj.P.Val ≤ 0.05) Gene Ontology terms for Monocytes for all PASC symptoms without adjustment for anti-spike antibody titers.

S3F Neutrophils NoTA: Table of significantly enriched (adj.P.Val ≤ 0.05) Gene Ontology terms for Neutrophils for all PASC symptoms without adjustment for anti-spike antibody titers.

S3G NK cells rest NoTA: Table of significantly enriched (adj.P.Val ≤ 0.05) Gene Ontology terms for NK cells resting for all PASC symptoms without adjustment for anti-spike antibody titers.

S3H Plasma cells NoTA: Table of significantly enriched (adj.P.Val ≤ 0.05) Gene Ontology terms for Plasma cells for all PASC symptoms without adjustment for anti-spike antibody titers.

S3I T cells CD4 mem. act. NoTA: Table of significantly enriched (adj.P.Val ≤ 0.05) Gene Ontology terms for T cells CD4 memory activated for all PASC symptoms without adjustment for anti-spike antibody titers.

S3J T cells CD4 mem. rest NoTA: Table of significantly enriched (adj.P.Val ≤ 0.05) Gene Ontology terms for T cells CD4 memory resting for all PASC symptoms without adjustment for anti-spike antibody titers.

S3K T cells CD8 NoTA: Table of significantly enriched (adj.P.Val ≤ 0.05) Gene Ontology terms for T cells CD8 for all PASC symptoms without adjustment for anti-spike antibody titers.

S3L T cells gamma delta NoTA: Table of significantly enriched (adj.P.Val ≤ 0.05) Gene Ontology terms for T cells gamma delta for all PASC symptoms without adjustment for anti-spike antibody titers.

S3M B cells mem. TA: Table of significantly enriched (adj.P.Val ≤ 0.05) Gene Ontology terms for B cells memory for all PASC symptoms with adjustment for anti-spike antibody titers.

S3N Macrophages M1 TA: Table of significantly enriched (adj.P.Val ≤ 0.05) Gene Ontology terms for Macrophages M1 for all PASC symptoms with adjustment for anti-spike antibody titers.

S3O Monocytes TA: Table of significantly enriched (adj.P.Val ≤ 0.05) Gene Ontology terms for Monocytes for all PASC symptoms with adjustment for anti-spike antibody titers.

S3P Neutrophils TA: Table of significantly enriched (adj.P.Val ≤ 0.05) Gene Ontology terms for Neutrophils for all PASC symptoms with adjustment for anti-spike antibody titers.

S3Q NK cells rest TA: Table of significantly enriched (adj.P.Val ≤ 0.05) Gene Ontology terms for NK cells resting for all PASC symptoms with adjustment for anti-spike antibody titers.

S3R Plasma cells TA: Table of significantly enriched (adj.P.Val ≤ 0.05) Gene Ontology terms for Plasma cells for all PASC symptoms with adjustment for anti-spike antibody titers.

S3S T cells CD4 mem. act. TA: Table of significantly enriched (adj.P.Val ≤ 0.05) Gene Ontology terms for T cells CD4 memory activated for all PASC symptoms with adjustment for anti-spike antibody titers.
S3T T cells CD4 mem. rest TA: Table of significantly enriched (adj.P.Val ≤ 0.05) Gene Ontology terms for T cells CD4 memory resting for all PASC symptoms with adjustment for anti-spike antibody titers.
S3U T cells CD8 TA: Table of significantly enriched (adj.P.Val ≤ 0.05) Gene Ontology terms for T cells CD8 for all PASC symptoms with adjustment for anti-spike antibody titers.
S3V T cells gamma delta TA: Table of significantly enriched (adj.P.Val ≤ 0.05) Gene Ontology terms for T cells gamma delta for all PASC symptoms with adjustment for anti-spike antibody titers.

Supplementary Table 4: DEGs for single-titer and term-elimination alternative models
Description: Description of sheets and columns
S4A PASC DEGs, term elim.: Table of significant DEGs (adj.P.Val ≤ 0.05) for all PASC symptoms and cell types, with specific terms dropped from the model (ModelVariant "No*").
S4B PASC DEGs, 1-titer adj.: Table of significant DEGs (adj.P.Val ≤ 0.05) for all PASC symptoms and cell types, with adjustment for single classes of anti-spike antibody titers (ModelVariant = "Serology_Ig*")

Supplementary Table 5: Marker gene enrichments in cell-type-specific DEGs
Description: Description of sheets and columns
S5A No Serology Adjustment: Cell type marker enrichment results for cell-type-specific DEGs with no serology adjustment
S5B Serology Adjustment: Cell type marker enrichment results for cell-type-specific DEGs with serology adjustment

Supplementary Table 6: Cell-type mapping for marker gene enrichments
Description: Description of sheets and columns
S6A DEG Cell Type mappings: Table of cell type mappings from LM22 reference to broad categories used to assemble lists of cell-type specific DEGs
S6B Marker Gene Cell Type Map: Table of cell type mappings used to annotate cell type marker genes to broad categories from the scientific literature
