# Supplementary material for: Molecular states during acute COVID-19 reveal distinct etiologies of long-term sequelae
Source: Nat Med. 2022 Dec 8;29(1):236–46. doi: 10.1038/s41591-022-02107-4 (PMC9873574; doi:10.1038/s41591-022-02107-4)
Supplement: Supplementary file 2 — Reporting Summary [file 41591_2022_2107_MOESM2_ESM.pdf]

nature portfolio

Corresponding author(s): Noam D. Beckmann
Alexander W. Charney

Last updated by author(s): Sep 7, 2022

Reporting Summary

Nature Portfolio wishes to improve the reproducibility of the work that we publish. This form provides structure for consistency and transparency in reporting. For further information on Nature Portfolio policies, see our Editorial Policies and the Editorial Policy Checklist.

Statistics

For all statistical analyses, confirm that the following items are present in the figure legend, table legend, main text, or Methods section.

n/a	Confirmed	
☐	☒	The exact sample size (*n*) for each experimental group/condition, given as a discrete number and unit of measurement
☐	☒	A statement on whether measurements were taken from distinct samples or whether the same sample was measured repeatedly
☐	☒	The statistical test(s) used AND whether they are one- or two-sided<br>*Only common tests should be described solely by name; describe more complex techniques in the Methods section.*
☐	☒	A description of all covariates tested
☐	☒	A description of any assumptions or corrections, such as tests of normality and adjustment for multiple comparisons
☐	☒	A full description of the statistical parameters including central tendency (e.g. means) or other basic estimates (e.g. regression coefficient) AND variation (e.g. standard deviation) or associated estimates of uncertainty (e.g. confidence intervals)
☐	☒	For null hypothesis testing, the test statistic (e.g. *F*, *t*, *r*) with confidence intervals, effect sizes, degrees of freedom and *P* value noted<br>*Give P values as exact values whenever suitable.*
☒	☐	For Bayesian analysis, information on the choice of priors and Markov chain Monte Carlo settings
☒	☐	For hierarchical and complex designs, identification of the appropriate level for tests and full reporting of outcomes
☐	☒	Estimates of effect sizes (e.g. Cohen's *d*, Pearson's *r*), indicating how they were calculated

Our web collection on statistics for biologists contains articles on many of the points above.

Software and code

Policy information about availability of computer code

Data collection

REDCap (10.0.25)
Epic Hyperspace (August 2019)
Epic Clarity (February 2020) on Oracle (18c Enterprise Edition Release 18.0.0.0.0)
Epic Caboodle (February 2020) on SQL server (Microsoft SQL Server 2016 (SP2-CU11) (KB4527378) - 13.0.5598.27 (X64))

Data analysis

R (4.0.3, 4.1.0, and 4.2.0)
Python (3.7.3)
MultiQC (1.9.dev0)
bcl2fastq (2.20.0)
STAR (2.7.3a)
fastqc (0.11.8)
Picard Tools (2.22.3)
Subread (1.6.3)
NGSCheckMate (https://github.com/DarwinAwardWinner/NGSCheckMate@45160a34acefa81e123cc2bd395b52937e66e0e2; git commit includes the custom modifications made to the software)
Bioconductor (3.12 - 3.15) (includes edgeR, limma, variancePartition, topGO, goseq, org.Hs.eg.db, BiocParallel)
WGCNA (1.69)
glmmLasso (1.5.1)
tidyverse (1.3.1)
ggplot2 (3.3.6)
data.table (1.14.2)
foreach (1.5.2)
doMC (1.3.8)
batchtools (0.9.15)

paralleIDist (0.2.6)
memoise (2.0.1)
cachem (1.0.6)
seriation (1.3.5)
treemap (2.4-3)
CIBERSORTx (software does not provide a version number)
Singularity (3.6.4)
GO-Figure! (https://gitlab.com/evogenlab/GO-Figure/-/tree/22ab3d84dbc0ea6d1121b82bc8838c78d2ea5cb3; software has no versioned releases, so we reference the git commit used)
NPX manager (2.1.0.224)
Clustergrammer (2)
Microsoft Excel (Office 365)

For manuscripts utilizing custom algorithms or software that are central to the research but not yet described in published literature, software must be made available to editors and reviewers. We strongly encourage code deposition in a community repository (e.g. GitHub). See the Nature Portfolio guidelines for submitting code & software for further information.

Data

Policy information about availability of data

All manuscripts must include a data availability statement. This statement should provide the following information, where applicable:

- Accession codes, unique identifiers, or web links for publicly available datasets
- A description of any restrictions on data availability
- For clinical datasets or third party data, please ensure that the statement adheres to our policy

All data, methods, and materials are available either in the main text, Methods, or Supplementary information, or via Synapse project ID syn35874390 (https://www.synapse.org/#!Synapse:syn35874390/). Validation data was obtained from previously published work.

Field-specific reporting

Please select the one below that is the best fit for your research. If you are not sure, read the appropriate sections before making your selection.

☒ Life sciences ☐ Behavioural & social sciences ☐ Ecological, evolutionary & environmental sciences

For a reference copy of the document with all sections, see nature.com/documents/nr-reporting-summary-flat.pdf

Life sciences study design

All studies must disclose on these points even when the disclosure is negative.

Sample size

Patients presenting to the Mount Sinai Health System (MSHS) between April and June of 2020 (i.e. the first wave of COVID-19 in New York City) were enrolled through daily manual review of new hospitalizations for COVID-19. During this time, as many patients as possible were enrolled each day given available manpower and resources. The patients enrolled on a given day were effectively a randomly sampled subset of all new patients with suspected COVID-19. Later, all enrolled subjects were asked to complete the checklist of post-acute symptoms (see methods).

COVID-19 cases (N = 495)
Blood samples with RNA-seq (N = 1392 samples, 567 subjects)
Blood samples with RNA-seq & serology (N = 1301 samples, 543 subjects)
COVID-19 subjects with checklists (N = 232)
COVID-19 subjects with RNA-seq & checklists (N = 165)
COVID-19 subjects with RNA-seq & checklists & serology (N = 158)

Using the RNASeqPower package (https://bioconductor.org/packages/release/bioc/html/RNASeqPower.html), we computed the expected power for small and large effect sizes (1.25-fold and 2-fold change respectively), using a coefficient of variation of 0.4 (the accepted typical value for human samples), an expected count of 21 (the 25th percentile of median gene counts in our data), N = 165 subjects split into equal-sized groups (symptom and no symptom), and an alpha (false positive rate) of 0.05. The expected power (true positive rate) under these conditions is 88% for a small effect size and >99% for a large effect size. The expected power for a typical symptom (lung problems, N = 34) was 72% for a small effect size and >99% for a large effect size. The expected power for the least-powered case for which DEGs were observed (pneumonia, N = 10) was 32% and >99% for small and large effect sizes respectively. This simplified power calculation ignores the longitudinal sampling of each subject and the hundreds of additional samples used for estimation of confounding factors, both of which would increase the effective power, and it does not take into account the degrees of freedom used to model the confounding factors or the cell-type-specific interaction model, which would somewhat decrease power.

Data exclusions

All COVID-19 cases were confirmed by a SARS-CoV-2 PCR test or serology within 2 weeks of initial sampling. All checklist answers from fully completed checklists were included.

RNA-seq samples that failed quality control were excluded, as well as RNA-seq samples with evidence of mislabeling for which correct sample labels could not be determined. QC and filtering of low-quality RNA-seq samples was performed according to pre-established standard procedures that were developed and tested on previous data sets prior to generation of the data.

ELISA data were excluded only if they showed clear aberrant titration values in dilution plates. In such cases, failed samples were re-run and the new values reported whenever possible. ELISA results corresponding to known mislabeled blood samples were also excluded.

Replication

Cell type deconvolution was replicated using 4 separate references. With no cohort of this size with comparable high-dimensional molecular data available for analysis, it was not possible to directly replicate or reproduce our other findings. We thus went with validation as described in the manuscript.

Randomization

Thorough randomization was performed for biological variables of interest before assignment to RNA-seq batches, with several batch control samples run in every batch to enable computational inference of batch effects (see methods). Relevant variables were controlled for in all linear mixed models.

ELISA samples were assigned to batches in near-real time as they were collected, with the exception that longitudinal samples from the same subject were assigned to the same batch whenever possible. Assay variation between ELISA batches was controlled using positive and negative controls for each antigen and secondary on each plate in every run. Titers are normalized negative controls. The CV for the positive controls was below 8% and very consistent over a 2-year period. ELISAs were benchmarked with two different CLIA tests and showed >99% sensitivity.

Blinding

This was not a randomized control trial; patients were recruited as they presented to the hospital, making blinding unneeded for this study. Data generation was inherently blinded to both acute-phase clinical information (including COVID-19 positive status and severity) and post-acute symptom information since these were compiled/collected and linked to samples only after RNA-seq and ELISA data had already been generated. Blinding was not used in the analysis because it is not generally possible to perform RNA-seq QC, differential expression testing, and related analyses in a blinded fashion.

Reporting for specific materials, systems and methods

We require information from authors about some types of materials, experimental systems and methods used in many studies. Here, indicate whether each material, system or method listed is relevant to your study. If you are not sure if a list item applies to your research, read the appropriate section before selecting a response.

Materials & experimental systems

n/a	Involved in the study
☐	☒ Antibodies
☒	☐ Eukaryotic cell lines
☒	☐ Palaeontology and archaeology
☒	☐ Animals and other organisms
☐	☒ Human research participants
☒	☐ Clinical data
☒	☐ Dual use research of concern

Methods

n/a	Involved in the study
☒	☐ ChIP-seq
☒	☐ Flow cytometry
☒	☐ MRI-based neuroimaging

Antibodies

Antibodies used

ELISA antibodies:
Goat Anti-Human IgM-AP SouthernBiotech, Cat. No. 2020-04, Lot No. L4206-Q408B, RRID AB_2795602, diluted 1/3000
Goat Anti-Human IgA-AP SouthernBiotech, Cat. No. 2050-04, Lot No. C5213-RI66P, RRID AB_2795704, diluted 1/4000
Goat Anti-Human IgG-AP SouthernBiotech, Cat. No. 2040-04, Lot No. B3919-NE80C, RRID AB_2795643, diluted 1/4500
Olink antibodies: Target 96 Inflammation panel

Validation

Antibodies for ELISA are quality tested by direct ELISA against standard reference reagents (from previous batches) on a panel of purified human immunoglobulins (IgM, IgG, & IgA) to ensure specificity to its respective isotype and minimal cross-reactivity with the remaining two isotypes.
ELISAs were benchmarked with two different CLIA tests and showed >99% sensitivity.
Validation data for antibodies used for Olink are available on the manufacturer's website: https://www.olink.com/content/uploads/2019/04/Olink-Inflammation-Validation-Data-v3.0.pdf

Human research participants

Policy information about studies involving human research participants

Population characteristics

Subjects ranged in age from 0 to 89 or more years old (mean = 62.25, standard deviation = 17.2). COVID-19 cases were all hospitalized, while SARS-CoV-2 negative controls were approximately half hospitalized and half healthy controls. The majority of subjects had at least one comorbidity. 325 subjects were male and 242 were female. Population characteristics of the subset of subjects who completed the PASC checklist are detailed in Table 1 and Supplementary Table 1A.

Differential expression testing was performed while controlling for variation in expression between male and female subjects. Differential expression testing was not performed for each sex individually because the sample size of subjects who completed the PASC checklist is insufficient for such analysis. Descriptive analysis of PASC symptoms includes tests of significant correlations between sex and PASC symptoms (Extended Data Figure 1). Randomization of samples into RNA sequencing batches was performed taking sex into account among other variables. Sex inferred from RNA-seq data was compared to sex recorded from clinical data to validate the correctness of sample labels.

Subject genders were not collected in this study.

Recruitment

COVID-19 cases and hospitalized controls were recruited as they presented in the hospital during the pandemic. All patients admitted to the Mount Sinai Health System were made aware of the research study by a notice included in their hospital intake packet. The notice outlined details of the specimen collection and planned research, and it provided instructions on how to opt-out of the study. Flyers announcing the study were also posted in the hospital and a video was run on the in-room hospital video channel. Given the monumental hurdles of consenting sick and infectious patients in isolation rooms, the Human Research Protection Program allowed for sample collection, which occurred at the time of clinical collection, prior to obtaining research consent. During or after hospitalization, research participants and/or their legally authorized representative provided consent to the research study, including genetic profiling for research and data sharing on an individual level. In those circumstances where consent could not be obtained (13.8% of subjects, 0% of subjects who completed the post-discharge checklist), data already generated could continue to be used for analysis purposes only when not doing so would have compromised the scientific integrity of the work. In this study of PASC, data from withdrawn and unconsented subjects was used only for quality control.

It is possible that some subsets of COVID-19 patients (e.g. severe vs. mild COVID-19) are more or less likely to consent than others, which could result in biased sampling. Healthy controls were recruited from research personnel working at the hospital during the pandemic. PASC checklists were filled only for subjects who chose to respond, potentially representing a self-selection bias. For example, patients experiencing no post-acute symptoms may have been more or less likely to respond than those experiencing symptoms. In principle, while this might bias the fraction of subjects with and without each symptom, this should not bias the differential expression and similar analyses between the two groups. (Instead, it will affect the power, which depends on the relative fractions of the symptom and no-symptom groups.)

Patients did not receive compensation for their participation in the study.

Ethics oversight

Human Research Protection Program at the Icahn School of Medicine at Mount Sinai (STUDY-20-00341)

Note that full information on the approval of the study protocol must also be provided in the manuscript.
